# Global transcriptome analysis of AtPAP2 - overexpressing *Arabidopsisthaliana* with elevated ATP

**DOI:** 10.1186/1471-2164-14-752

**Published:** 2013-11-01

**Authors:** Feng Sun, Chao Liang, James Whelan, Jun Yang, Peng Zhang, Boon Leong Lim

**Affiliations:** 1 School of Biological Sciences, the University of Hong Kong, Pokfulam, Hong Kong, China; 2 Australian Research Council Centre of Excellence in Plant Energy Biology, University of Western Australia, Crawley, WA 6009, Australia; 3 Botany Department, School of Life Science, La Trobe University, Bundoora 3086 Victoria, Australia; 4 National Laboratory of Plant Molecular Genetics and National Center for Plant Gene Research (Shanghai), Institute of Plant Physiology & Ecology, Shanghai Institutes for Biological Sciences, Chinese Academy of Sciences, 300 Fenglin Road, Shanghai 200032, China; 5 Partner State Key Laboratory of Agrobiotechnology, The Chinese University of Hong Kong, Shatin, Hong Kong, China

**Keywords:** Chloroplast, Mitochondria, LHC, Redox, Photosystem, Transcriptomes

## Abstract

**Background:**

AtPAP2 is a purple acid phosphatase that is targeted to both chloroplasts and mitochondria. Over-expression (OE) lines of AtPAP2 grew faster, produced more seeds, and contained higher leaf sucrose and glucose contents. The present study aimed to determine how high energy status affects leaf and root transcriptomes.

**Results:**

ATP and ADP levels in the OE lines are 30-50% and 20-50% higher than in the wild-type (WT) plants. Global transcriptome analyses indicated that transcriptional regulation does play a role in sucrose and starch metabolism, nitrogen, potassium and iron uptake, amino acids and secondary metabolites metabolism when there is an ample supply of energy. While the transcript abundance of genes encoding protein components of photosystem I (PS I), photosystem II (PS II) and light harvesting complex I (LHCI) were unaltered, changes in transcript abundance for genes encoding proteins of LHCII are significant. The gene expressions of most enzymes of the Calvin cycle, glycolysis and the tricarboxylic acid (TCA) cycle were unaltered, as these enzymes are known to be regulated by light/redox status or allosteric modulation by the products (e.g. citrate, ATP/ADP ratio), but not at the level of transcription.

**Conclusions:**

AtPAP2 overexpression resulted in a widespread reprogramming of the transcriptome in the transgenic plants, which is characterized by changes in the carbon, nitrogen, potassium, and iron metabolism. The fast-growing AtPAP2 OE lines provide an interesting tool for studying the regulation of energy system in plant.

## Background

Purple acid phosphatases (PAPs) catalyze the hydrolysis of phosphoric acid esters and anhydrides [1]. In higher plants, PAPs are mostly related to the Pi response [2,3]. The only PAP that has been shown to affect carbon metabolism is AtPAP2, which is targeted to both chloroplasts and mitochondria by an additional transmembrane motif at the C-terminus compared to other related proteins [4]. PAPs with a transmembrane motif at their C-termini are conserved in green plants, including the smallest free-living photosynthetic eukaryote, *Ostreococcus tauri*[5]. Transgenic *Arabidopsis thaliana* overexpressing AtPAP2 grew faster, produced more seeds and contained higher leaf sucrose content (up to 30%) [6]. Transgenic *Camelina sativa* overexpressing AtPAP2 also grew faster and produced more seeds [7]. The pleiotropic growth-promoting effect of AtPAP2 is dependent on its C-terminal dual-targeting sequence [6].

Chloroplasts and mitochondria are two key organelles involved in energy metabolism in plant cells and how AtPAP2 affects the biology of these two organelles and supplies more energy for growth remains unknown. To study the impact of AtPAP2 overexpression on the energy status of plants, the levels of ATP and ADP in the leaves of 20-day-old AtPAP2 OE Arabidopsis were measured and compared with those of WT plants. The transcriptomes of leaves and roots were also compared. AtPAP2 overexpression resulted in a widespread changes of the transcriptome in the transgenic plants, which may reflect the impact of changes in energy supply that feed back to alter transcriptional programmes.

## Results

### AtPAP2 OE lines contain elevated levels of ATP

To determine if the overexpression of AtPAP2 resulted in alteration in metabolites, LC-MS/MS analysis and bioluminescent based assays were carried out to measure the amount of ATP and ADP. As shown in Table 1, the leaves of AtPAP2 OE lines contained higher levels of ATP and ADP compared with the WT. In contrast, the AtPAP2 T-DNA line contained similar levels of ATP and ADP to the WT. The ATP/ADP ratios among these lines were also unchanged. The levels of ATP and ADP in the WT are similar to those measured in the other studies [8].

**Table 1:** **ATP/ADP contents in 20-day-old *Arabidopsis thaliana *** **leaves**

**Methods**	**Lines**	**ATP**	**ADP**	**ATP + ADP**	**ATP/ADP**
		**(nmol/gFW)**	**(nmol/gFW)**	**(nmol/gFW)**	
LC-MS/MS (n = 3 ~ 4)	WT	34.17 ± 5.79^a^	26.07 ± 0.79^a^	60.24 ± 6.44^a^	1.31 ± 0.19^ab^
	T-DNA	32.14 ± 1.47^a^	28.72 ± 0.50^a^	60.86 ± 1.94^a^	1.12 ± 0.03^a^
	OE7	47.87 ± 4.36^b^	32.03 ± 3.33^a^	79.90 ± 6.55^b^	1.50 ± 0.15^b^
	OE21	53.98 ± 3.09^b^	40.73 ± 5.31^b^	94.71 ± 7.48^b^	1.34 ± 0.15^ab^
Bioluminescent assay (n = 5)	WT	25.26 ± 3.88^a^	14.81 ± 2.12^ab^	40.07 ± 5.38^a^	1.71 ± 0.22^a^
	T-DNA	21.47 ± 2.24^a^	13.10 ± 1.51^a^	34.57 ± 3.71^a^	1.64 ± 0.05^a^
	OE7	32.72 ± 2.93^b^	20.07 ± 1.57^c^	52.79 ± 4.13^b^	1.63 ± 0.11^a^
	OE21	32.73 ± 3.29^b^	17.71 ± 1.23^bc^	50.45 ± 4.04^b^	1.85 ± 0.16^a^

Statistical differences (P < 0.05) in the same column for each line were based on one-way ANOVA analysis followed by Tukey’s Honestly Significant Differences (HSD) test using statistical program IBM SPSS 19. Within each column, the values marked by different letters (a, b, c) are significantly different (P < 0.05). The data were reproducible in at least 3 independent experiments.

### Identification of genes differentially expressed in the AtPAP2 OE plants

**Figure 1:**
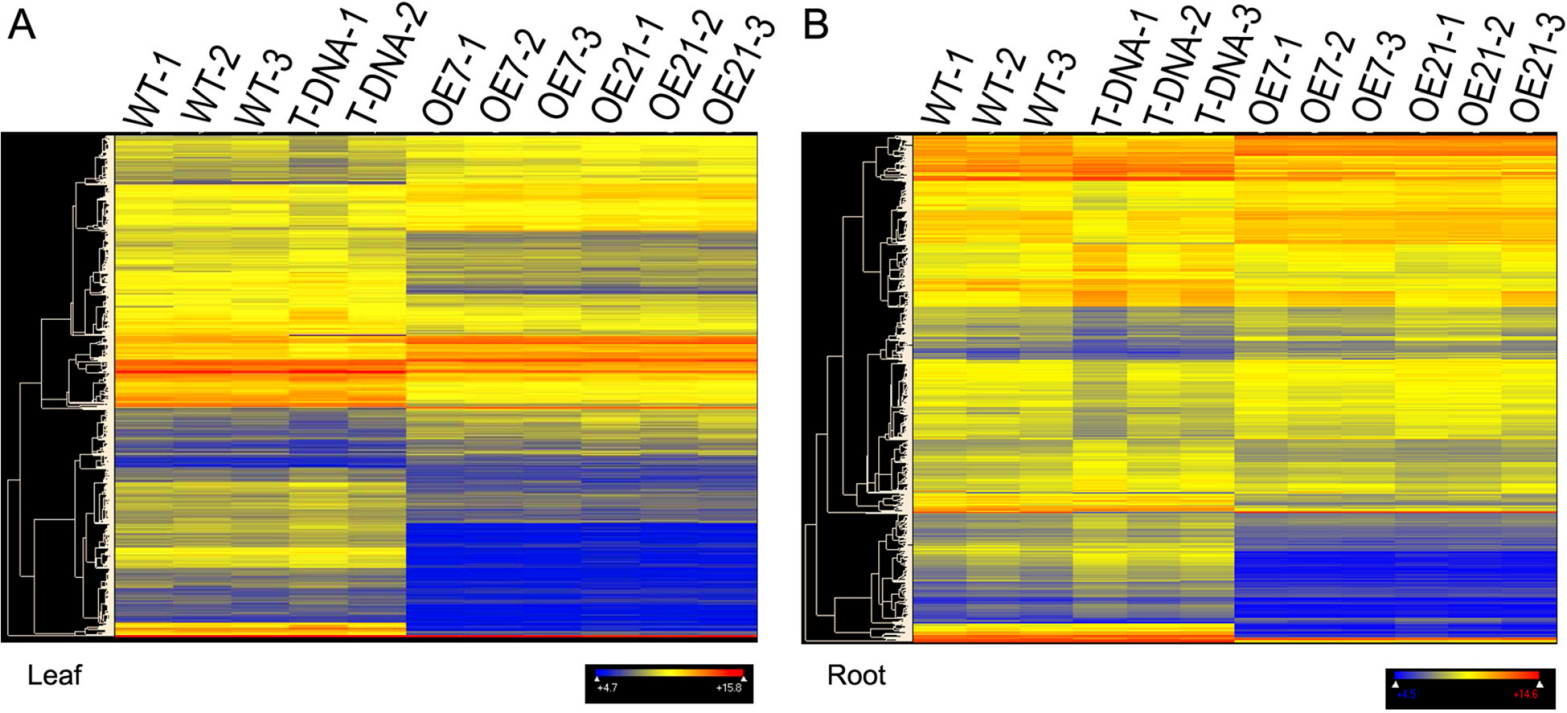
**Transcriptional profiling of microarray data in leaves and roots.** ArrayStar 3.0 was used to perform heat map clustering. Three replicates (T-DNA line had 2 replicates in the leaves) from the leaves **(A)** and roots **(B)** of 20-day-old Arabidopsis were analyzed. The color scale indicates the expression levels. Blue indicates low expression, while red stands for high expression.

To identify the molecular events associated with the fast growth phenotype of AtPAP2 OE lines, gene expression profiles from soil-grown 20-day-old WT, AtPAP2 T-DNA line and AtPAP2 OE lines were analyzed with NimbleGen cDNA Arrays at the middle of day under long day growth conditions (Figure 1). The lines at this stage did not show any differences in size, leaf number and appearance. There were 30361 genes represented on the chip for leaf RNA analysis and 37118 genes on the chip for root RNA analysis. Each line had three biological replicates and the average hybridization signals detected in each line were normalized and compared with the signal intensities in the WT. Pair-wise plots revealed good agreement between biological replicates (Figure 1). Genes with mean signal intensities less than 100 in all four lines (WT, T-DNA, OE7 and OE21 lines) were excluded from analysis. Therefore, only 28817 genes from leaves and 31148 genes from roots were analyzed for differential expression. Genes with mean signal intensities that differed significantly were filtered by a 1.5-fold change (FC) and P <0.05 in a Student’s *t*-test in both OE7 and OE21 lines when compared with the WT. Of the significantly responsive transcripts, 3308 genes (11.5%) in the leaves and 2313 genes (7.4%) in the roots of both AtPAP2 OE lines were altered compared to WT (Additional files 1 and 2). The overall view of the altered genes presented in a heat map (Figure 1) revealed that transcript abundance of most genes were downregulated in both leaves and roots, with fewer genes displaying an increase in transcript abundance, 2051 out of 3308 transcripts were decreased in abundance in leaves, while 1631 out of 2313 transcripts were decreased in abundance in roots.

**Figure 2:**
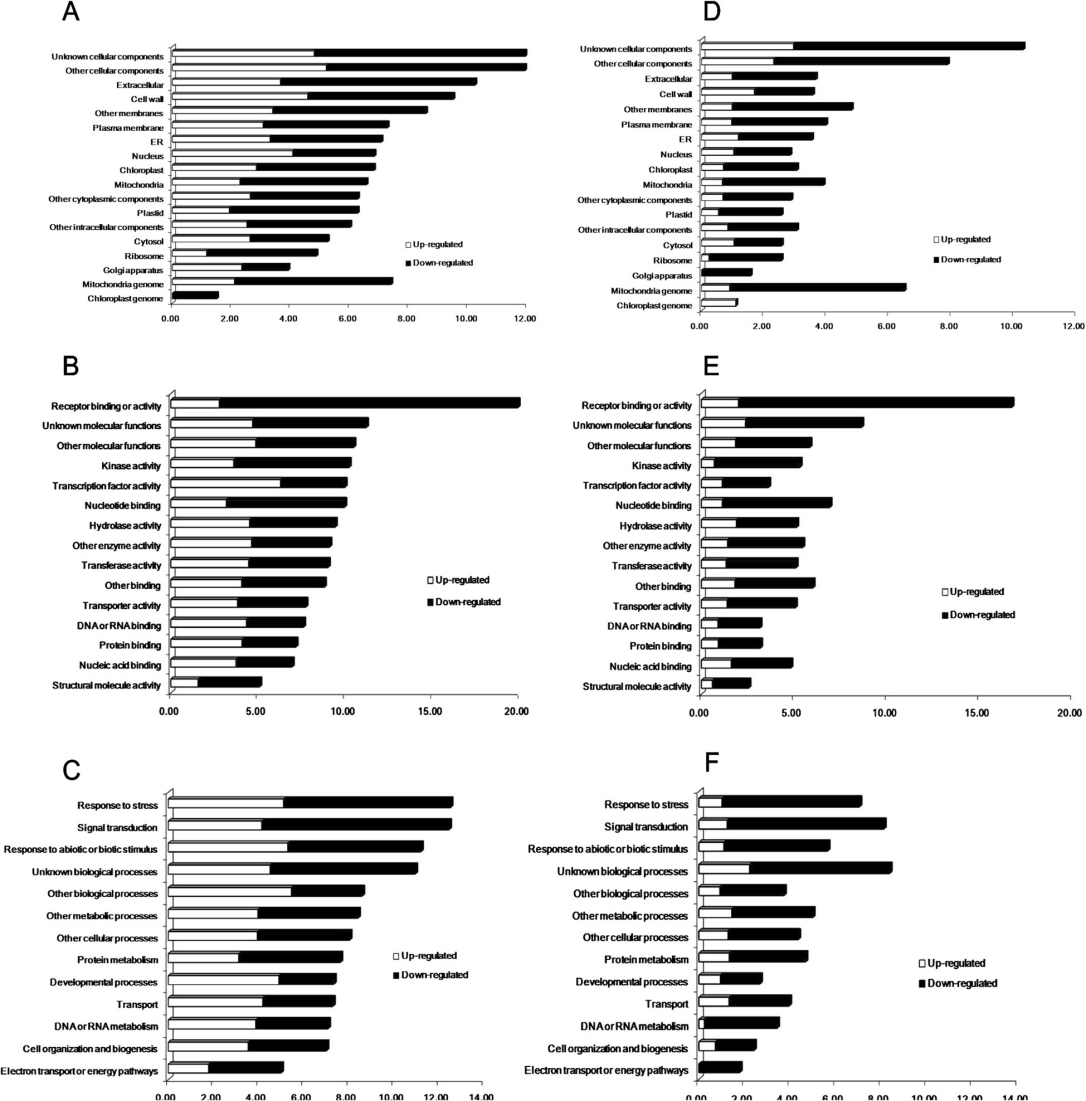
**GO functional analysis of differentially expressed genes in leaves and roots.** Functional category of differentially expressed genes in the leaves **(A,B,C)** and roots **(D,E,F)** in both AtPAP2 OE lines compared with the WT. Genes were annotated as “Cellular component” **(A,D)**, “Molecular function” **(B,E)** and “Biological processes” **(C,F)** (1.5-fold change, P < 0.05).

Functional category enrichment evaluation was performed using gene ontology (GO) analysis in TAIR (Figure 2). Analysis of the ratio of genes (Number of significantly changed genes/number of total genes in each category) indicated different expression patterns in each cluster. In general, more genes in leaves exhibited significant changes than in roots (Figure 2). This correlates with the more drastic phenotypic changes in leaves than in roots. This is reasonable, because overexpression of AtPAP2 could have direct impacts on both chloroplasts and mitochondria in leaves, but only on mitochondria in roots, although an effect on plastids cannot be excluded. In the “Cellular component” cluster, there are more downregulated than upregulated transcripts in most of the categories in both leaves and roots. The only exception is “Nucleus” in leaves, which has more upregulated transcripts, possibly indicating changes in regulators of gene transcription (Figure 2A). In the “Molecular function” cluster, the category “Receptor binding or activity” displayed most changes, where most transcripts were downregulated. Interestingly, there are more upregulated than downregulated transcripts in the category of “transcription factor activity” in the leaves (Figure 2B). In the GO clustering of “Biological processes” in the leaves, the numbers of upregulated transcripts in the “Developmental processes”, “DNA and RNA metabolism” and “Transport” are greater than the numbers of downregulated transcripts, which could correlate with the fast-growing phenotype of the AtPAP2 OE lines (Figure 2C). This was not observed in the roots (Figure 2F), where all GO categories had more downregulated transcripts (Figure 2D-F). In addition, GO annotation also showed very few gene changes in the AtPAP2 T-DNA line compared to the WT (data not shown), which is consistent with the WT-like phenotype of the AtPAP2 T-DNA line, implying a redundant function of AtPAP2 with other protein homologs in the genome.

**Figure 3:**
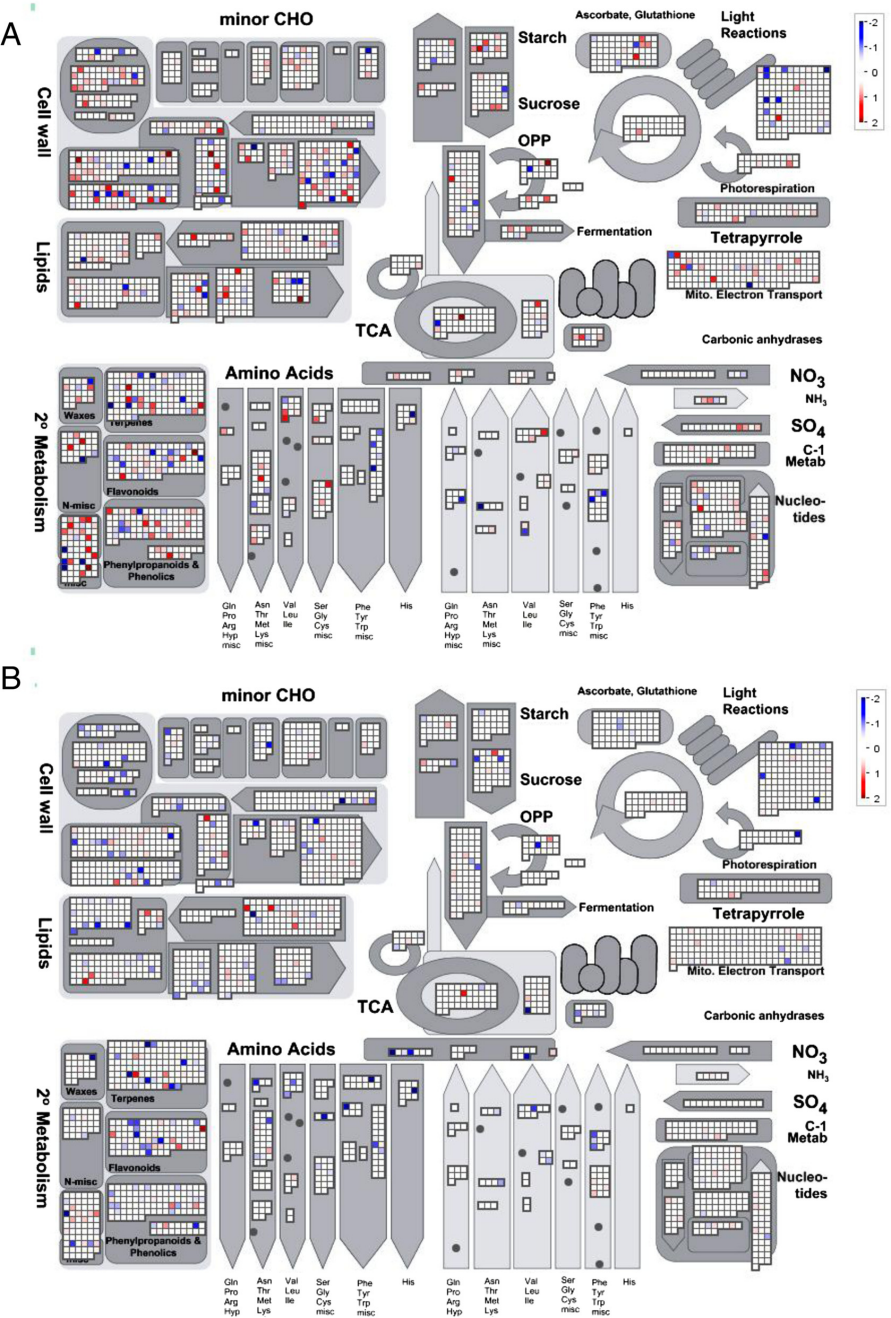
**Changes in transcripts associated with general metabolism.** Compared with the WT, genes significantly up- and downregulated (1.5-fold change and P < 0.05) in the leaves **(A)** and roots **(B)** of AtPAP2 OE lines are visualized by MapMan and are indicated in red and blue, respectively. Scale bars display log2 fold changes.

Data for specific groups of genes were extracted and studied using the MapMan hierarchical ontology software (Figure 3 and Additional files 3, 4, 5, 6 and 7) [9] (http://www.gabipd.org/projects/MapMan/; Ath_AGI_TAIR9; Additional file 1 and 2). Again, more transcripts in leaves exhibited significant changes than in roots, including transcripts encoding proteins involved in carbohydrate metabolism (10.1% in leaves versus 3.4% in roots), cell wall metabolism (17.7% in leaves versus 3.5% in roots), glycolysis, mitochondria electron transport, ATP synthesis and the TCA cycle (9.3% in leaves versus 3.0% in roots), amino acid synthesis (11.6% in leaves versus 7.1% in roots) and lipid metabolism (8.0% in leaves versus 5.1% in roots). In addition, many genes encoding proteins associated with development (11.4% in leaves and 4.6% in roots), transcription (10.3% in leaves versus 4.4% in roots), protein modification and degradation (11.2% in leaves versus 5.6% in roots), stress (19.2% in leaves versus 12.4% in roots) and redox regulation (23.2% in leaves versus 7.6% in roots) exhibited remarkable changes (Additional files 3, 4, 5, 6 and 7). Thus, widespread reprogramming of the transcriptome in the AtPAP2 OE plants corresponded with their fast-growing phenotypes. A complete list of altered genes, including their putative function, can be found in Additional file 1 (leaves) and Additional file 2 (roots).

### Energy-harvesting system in leaves

When the expression profiles of the energy-harvesting system in the source leaves were compared, transcript levels of genes encoding PS I core proteins, PS II core proteins, LHC I proteins were not significantly altered (FC ≥ ±1.5) (Additional file 8a). In contrast, transcript levels of several genes encoding LHCII proteins, including *Lhcb* 1.4 (AT2G34430, FC ≤ 0.47), *Lhcb* 2.2 (AT2G05070, FC ≤ 0.06), *Lhcb* 2.3 (AT3G27690, FC ≤ 0.66), *Lhcb* 4.2 (*CP29*, AT3G08940, FC ≤ 0.38) and *Lhcb* 6 (*CP24*, AT1G15820, FC ≤0.44), were decreased (FC ≤ 0.66) in the AtPAP2 OE lines. The transcript levels encoding other components in the electron flow chain, including cytochrome b_6_f complex, ferridoxin (Fd), plastocyanin (PC), NADPH dehydrogenase (NDH) complex, were mostly unaltered, except one of the two ferredoxin-NADP + reductases (*FNR2,*AT1G20020, FC ≤ 0.57), *FdC2* (AT4G14890, FC ≤ 0.57) [10] and *NdhO* (AT1G74880, FC ≤ 0.66) [11], which was suppressed in the OE lines. Only two transcripts were upregulated in the OE lines, including *PGR5-like B (PGRL1B,* AT4G11960, FC ≥ 2.30), and *cyt c6a* (AT5G45040, FC ≥ 2.50) [12]. All these changes reflected a reprogramming of energy harvest and electron transfer in the photosystems. Note that organelle-encoded genes are not poly-adenylated and therefore their cDNAs were not labeled in this study.

### Redox regulated proteins in leaves and roots

In leaf, electrons excited by sunlight are the ultimate source of reducing equivalents in plants. Electron flow generated from the photosystems is used to reduce Fd, which in turn can be used for reduction of NADP to NADPH by FNR, for reduction of thiodoxins (Trx) by ferredoxin:thioredoxinreductase (FTR), for reduction of nitrite to ammonium by ferredoxin-nitrite reductase (NiR) [13] and for reduction of 2-oxoglutarate and glutamate to two glutamines by glutamate synthase (GOGAT). In the leaves and roots of the OE lines, there were no changes in the transcript abundance of four *Fd* genes, two *FTR* genes, nine *Trx* genes (*Trx f1-2, m1-4, Trx x, Trx y1-2*), or most enzymes regulated by Trx (GAPD1-3, SBPase, PRK, RCA, NADP-MDH) (Additional file 8b) [14]. In leaf, but not in root, the transcripts of two electron carriers *FNR2* and *FdC2* which play roles in photosynthesis, were downregulated. Interestingly, the transcripts of *NiR* (AT2G15620, FC ≤ 0.63) was downregulated in the roots, whereas *G6PD4* (AT1G09420) were downregulated specifically in both leaves (FC ≥ 4.38) and roots (FC ≥ 1.95), whereas the transcript of *ATO*1 (AT2G35010), a threodoxin in mitochondria, was upregulated in leaves (FC ≥ 1.55) but was downregualted in roots (FC ≤ 0.03), respectively.

### Calvin cycle, starch and sucrose synthesis in leaves

Transcript abundance for the key enzyme of the Calvin cycle, a transketolase (AT2G45290, FC ≥ 1.97), was induced in the leaves of AtPAP2 OE lines. Upregulation of the transcript implied an activated Calvin cycle activity and possible enhanced output of carbon skeletons for sucrose synthesis in the cytosol. AtPAP2 OE lines exhibited increased leaf sucrose content, a higher expression level of SPS protein and a higher SPS activity in leaves [6]. Among the four *SPS* genes in Arabidopsis, *AtSPS2F* (AT5G11110) exhibited a significant change in both leaves (FC ≥ 1.85) and roots (FC ≥ 1.73) of AtPAP2 OE lines (Additional files 8 and 9). Regarding sucrose cleavage enzymes that import sucrose in the sink tissues, two out of six sucrose synthases, *SuSy1* (AT5G20830, FC ≥ 1.90) and *SuSy3* (AT4G02280, FC ≥ 1.75) were upregulated in leaves, whereas transcripts of a gene for cell wall invertase (AT3G13790, FC ≤ 0.45) was significantly downregulated in the leaves of the OE lines (vs. WT). These results indicated an alteration of the gene expression pattern in sucrose metabolism. The transcript abundance of two key genes encoding enzymes in starch synthesis, plastid phosphoglucomutase (*PGM*, AT5G51820, FC ≤ 0.53) and ADP-glucose pyrophosphatase small subunit 2 (AT1G05610, FC ≥ 1.74) were altered in the leaves. In addition, the transcript abundance of three starch degradation enzymes: water dikinase (AT4G24450, FC ≥ 1.68), α-amylase 3 (AT1G69830, FC ≥ 3.0) and glucanphophorylase (AT3G29320, FC ≤ 0.60), were significantly changed. Taken together, AtPAP2 OE lines exhibited altered gene expression patterns for sucrose and starch metabolism.

### Glycolysis, the TCA cycle and the electron transport chain in mitochondria

Except for the upregulation of a cytosolic pyruvate kinase (AT5G56350, FC ≥ 1.64) in leaves and a downregulation of a pyruvate kinase (AT3G49160, FC ≤ 0.55) in roots, the expression levels of all of the genes of cytosolic enzymes involved in glycolysis were unaltered in leaves and roots (Additional files 8e and 9d). Likewise, the expression levels of all genes in the TCA cycle, except for the upregulation of a citrate synthase (CS)-like gene (AT2G11270, FC ≥ 5.30 in leaves and FC ≥ 2.53 in roots), were unchanged in the leaves and roots of both AtPAP2 OE lines (Additional files 8f and 9e). Regarding the respiratory chain in mitochondria, only components of Complex I, but not components of Complexes II, III, IV, V, UBQ and cytochrome c oxidase (COX) biogenesis, were altered in both leaves and roots (Additional file 10).

### Cell wall synthesis

Thirty percent or more of cellular carbohydrate metabolism is consumed by the synthesis of wall components and cell shape morphogenesis [15]. Enhanced ATP production in source leaves of AtPAP2 OE lines could lead to a higher supply of sucrose for cell wall synthesis to support plant growth. AtPAP2 OE lines also exhibited significant changes in the expressions of genes encoding polygalacturonase, pectinesterase, cellulase and cellulose synthase (Additional files 8d and 9c). Transcripts from 87 genes (17.7%) in leaves (Additional file 1d) and 18 genes (3.5%) in roots (Additional file 2d) were altered, which correlated with a higher growth rate in leaves than in roots, arising from enhanced sucrose synthesis in leaves. Transcript of a gene encoding a protein similar to cellulose synthase (*ATCSLA01,* AT4G16590, FC ≥ 6.69) in the cellulose synthesis was induced up to 11-fold in the leaves of AtPAP2 OE lines. In addition, transcript levels of genes encoding expansin or expansin homologs (AT4G17030, FC < 0.35; AT1G69530, FC < 0.44; AT5G02260, FC < 0.59; AT2G20750, FC < 0.60; AT3G29365, FC < 0.62; AT1G20190, FC > 1.53; AT4G38400, FC > 1.91) and xyloglucan endotransglycosylase (AT4G25810, FC ≤ 0.33; AT4G14130, FC ≥ 2.50) were also significantly upregulated in abundance. These alterations reflected an active state for cell wall growth and reorganization in leaves.

### Nitrogen and amino acid metabolism

Faster plant growth and higher seed yield requires greater supply of nitrogen or re-allocation of available nitrogen sources. Plants obtain nitrogen via ammonium or nitrate transporters in roots. Nitrate is reduced to ammonium by two biochemical steps, nitrate reduction and nitrite reduction. The expression of the only nitrite reductase gene, *NiR* (AT2G15620), was significantly downregulated in the leaves (FC ≤ 0.63), Nitrate transporters, including *AtNRT1.1* (AT1G12110, FC ≤ 0.55 in the leaves) [16], *AtNRT1.2* (AT1G69850, FC ≤ 0.62 in the leaves and FC ≤ 0.58 in the roots) [17] were downregulated in the leaves and roots of both OE lines. Interestingly, a high affinity ammonium transporter (*AMT1;2*, AT1G64780, FC ≥ 3.12), was significantly induced in leaves. The reduced Fd generated from photosynthesis activates NiR activity posttranslationally; therefore, its lower mRNA expression in the OE lines could result from negative feedback by higher specific activities. If the NiR activity is indeed higher in the OE lines, more ammonium, but less nitrate, will be transported to the leaves. This could result in the upregulation of ammonium transporters, but downregulation of nitrate transporters in the leaves.

Amino acids serve as precursors of metabolites or intermediates for the stress response [18]. Noticeably, there was a clear tendency of repressed expression of genes associated with biosynthesis of the aspartate family amino acids (Asn, Asp, Lys, Met, Thr, Ile) in the roots of both OE lines. In the leaves, except for genes of enzymes involving in the biosynthesis of homoserine, genes for Pro, Cys, Ser and two genes for Met synthesis, were upregulated; all other genes involved in Trp and Lys synthesis, and Arg, Trp, and Ile degradation were downregulated.

The aromatic amino acids (AAA) metabolic pathway covers the synthesis of Trp, Phe and Tyr [19]. Nearly all the genes involved in this pathway were downregulated in both leaves and roots, suggesting a decreased transcriptional expression activity in the AAA metabolic pathway. In addition, a cytosolic NADP^+^-isocitrate dehydrogenase (*ICDH*, AT1G65930, FC ≤ 0.43) responsible for 2-oxoglutarate production in amino acid synthesis [20] was also downregulated in leaves.

### Potassium and iron uptake

The major potassium channel in leaves (AKT2/3) is positively regulated by a kinase (CIPK23) and negatively regulated by a phosphatase (AIP1) [21]. Expression of *CIPK23* (AT1G30270, FC ≥ 1.60) was significantly upregulated in the leaves of the OE lines (Additional file 8h). Regarding Fe uptake, one out of three Fe (II) transporters (*IRT3*, AT1G60960, FC ≥ 2.03) was significantly upregulated in leaves, and the expression of four out of eight ferric reductases including *FRO1* (AT1G01590, FC ≥ 1.74; ), *FRO4* (AT5G23980, FC ≥ 6.87), *FRO3* (AT1G23020, FC ≤ 0.37) and *FRO8* (AT5G50160, FC ≤ 0.60) changed significantly (Additional file 8i) whereas in the roots, transcripts of *FRO3* (FC ≤ 0.62) and *FRO8* (FC ≥ 1.57), were significantly altered in the roots (Additional file 9h).

### Secondary metabolism

Sixty-three out of 395 genes involved in secondary metabolism in leaves were affected (Additional file 1). These include genes involved in phenylpropanoid and flavonoid biosynthesis (i.e. *PAL2, PAL3, CHS, UGT71D1, CYP706A4, CYP706A5, UF3GT, DFR, ATNIC1, CAD5*) and genes involved in phenols, glucosinolates, wax, and isoprenoids synthesis and degradation.

### Transcription factors

Genes encoding transcription factors (TFs) constitute 5 to 7% of the Arabidopsis genes [22]. The Arabidopsis genome encodes at least 1550 TFs, classified into more than 50 families [23]. In this study, a larger amount of TFs were differentially expressed in the leaves (233 from 45 families) than in the roots (103 from 31 families) of AtPAP2 OE plants (Additional file 11). The number of upregulated TFs (6.3%) was higher than the number of downregulated TFs (3.7%) in the leaves; however, in roots, there were fewer upregulated genes (1.8%) than downregulated genes (2.5%) (Figure 2). In leaf, the transcriptional repressor NF-YA5, was up-regulated (AT1G54160, FC ≥ 1.74) at the transcriptional level. NF-YA5 could specifically bind to miR169, which targets mRNAs for cleavage or translational repression at multiple cellular processes [24]. The mRNA of the transcriptional activators such as MYB58 (AT1G16490, FC ≥ 2.00) in the lignin biosynthetic pathway [25], a FLOWERING BHLH transcriptional activator (AT4G09180, FC ≥ 1.74) control expression of the photoperiodic flowering [26], were also found with increased transcripts abundance in the leaves. These repressors or activators might affect targeted gene expression to some extent at the transcriptional level.

### Nucleus-encoded chloroplastic and mitochondrial proteins

As AtPAP2 is targeted to the chloroplasts and mitochondria, the transcripts of genes encoding proteins of the “Chloroplast” and “Mitochondria” categories of TAIR were examined. About 6.8% genes in the “Chloroplast” and 6.6% genes in the “Mitochondria” of leaves and 3.9% genes in the roots “Mitochondria” transcripts were significantly changed. Among ~1500 nucleus-encoded proteins identified in chloroplasts by proteomics studies [27], more transcripts were significantly changed in the leaves (91 or 6.1%) than in the roots (43 or 2.9%) (Additional file 12). Among ~650 nucleus-encoded proteins identified in mitochondria by proteomics studies [28,29], again, more transcripts were significantly altered in leaves (37 or 5.7%) than in roots (10 or 1.5%) (Additional file 12).

### Verification of candidate genes by real-time RT-PCR

To confirm the accuracy of the microarray data, real-time RT-PCR analysis was carried out on randomly selected genes from leaves. Candidate genes selected were: a C2 domain-containing protein (AT3G60950, FC ≥ 62.0), a member of the receptor kinase-like protein family (AT3G24660, FC ≥ 1.32), a phosphatidylinositol 3- and 4-kinase family protein (AT5G24240, FC ≥ 10.2), a tyrosine specific protein phosphatase family protein (AT1G05000, FC ≥ 1.53) and a protein kinase family protein (AT1G28390, FC ≥ 2.38). Gene expression values from real-time RT-PCR of the five genes were also compared to their values from the microarray data. The expression of each gene was consistent between the microarray and real-time RT-PCR results (Additional files 13 and 14).

### Microarray data is highly correlated with the physiology of AtPAP2 OE lines

**Figure 4:**
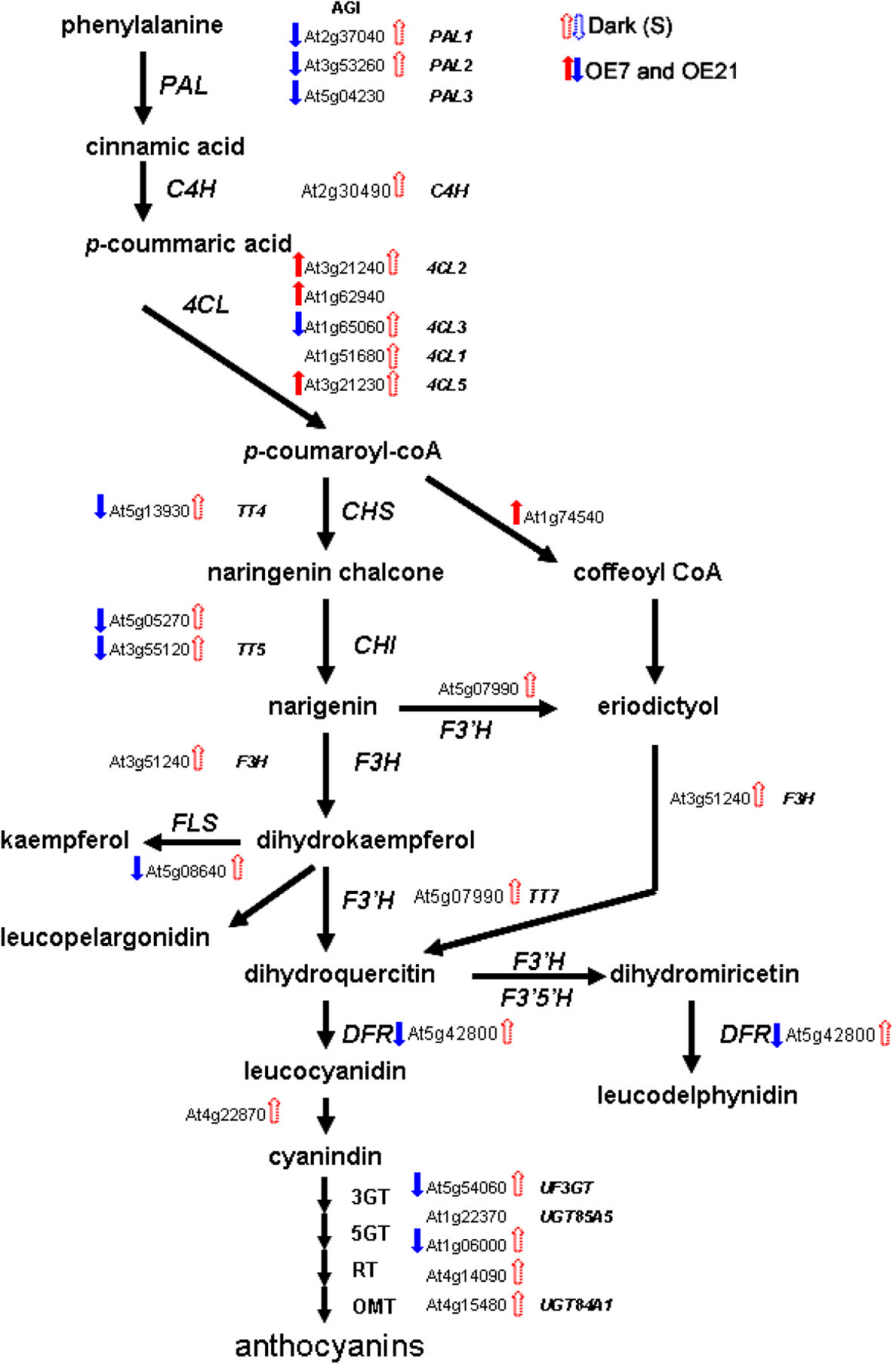
**Genes of the anthocynain biosynthesis pathway are differentially affected by exogenous sucrose and endogenous sugars (AtPAP2 OE lines).** Genes that were significantly altered (*P <* 0.05) are shown (arrows). Solid arrows indicate gene expression in OE lines while empty arrows showed the sucrose-modulated genes in dark grown WT seedlings (Dark (S)). Abbreviations are as follows: AGI, *Arabidopsis* Genome Initiative; *C4H*, cinnamate-4-hydroxylase; *4CL*, 4-coumaroyl-CoA synthase; *CHI*, chalcone isomerase; *DFR*, dihydroflavonol 4-reductase; *F3H*, flavanone 3-hydroxylase; *F3′H*, flavonoid 3′-hydroxylase; *F3′5′H*, flavonoid 3′5′-hydroxylase; *FLS*, flavonol synthase; *3GT*, 3-glucosyl transferase; *5GT*, 5-glucosyl transferase; *OMT*, O-methyl transferase; *PAL*, phenylalanine ammonia-lyase; *RT*, rhamnosyl transferase; *TT4*, Transparent testa 4; *TT5*, Transparent testa 5; *TT7*, Transparent testa 7; *UF3GT*, UDP-Glc: flavonoid 3-*O*-glucosyltransferase.

High exogenous sucrose induces anthocyanin biosynthesis in Arabidopsis [30]. In the AtPAP2 OE plants, most genes in the anthocyanin biosynthesis pathway were repressed (Figure 4). The key pathway gene *dihydroflavonol 4-reductase (DFR)* and downstream *UF3GT* decreased 2-fold compared to WT. Other genes in the anthocyanin pathway also had attenuated gene expression in the OE lines. Thus, these microarray data predict opposite effects of endogenous and exogenous sucrose on anthocyanin biosynthesis.

To examine the correlation between the microarray data and phenotypes, the levels of anthocyanin production in various lines were examined on sugar-treated MS plates. AtPAP2 T-DNA insertion line had accumulated more anthocyanin than WT. In contrast, AtPAP2 OE7 line showed remarkably less purple color under the same treatment (Additional files 15 and 16). Mannitol and sorbitol are reduced forms of glucose and are not efficiently metabolized by plants [30]. Higher concentration of mannitol and sorbitol (8% and 9%; w/v) were added to the MS medium. These sugars also showed an anthocyanin background but there were few differences among the WT, AtPAP2 T-DNA line and AtPAP2 OE lines (Additional file 16). A semi-quantitative reverse transcription-PCR (RT-PCR) was employed to verify the transcriptional expression of genes involved in the pathway. In high sucrose (6%, w/v) MS medium, both WT and T-DNA lines exhibited strong induction of *DFR* and P*roduction of anthocyanin pigment 1,* (*PAP1*) [30] whereas, the AtPAP2 OE7 line showed only weak induction of these 2 key genes (Additional file 17). Hence, the microarray data highly correlates with the physiology of the OE lines.

## Discussion

Metabolomics analysis showed that AtPAP2 OE lines contained higher level of ATP (Table 1) and higher malate, citrate, fumarate and sucrose [6]. The fast growth, high seed yield and high sucrose phenotypes imply that the energy harvesting system of the OE lines may be more efficient, as the cell sizes and cell densities of the OE lines did not differ significantly compared to WT (data not shown).

**Figure 5:**
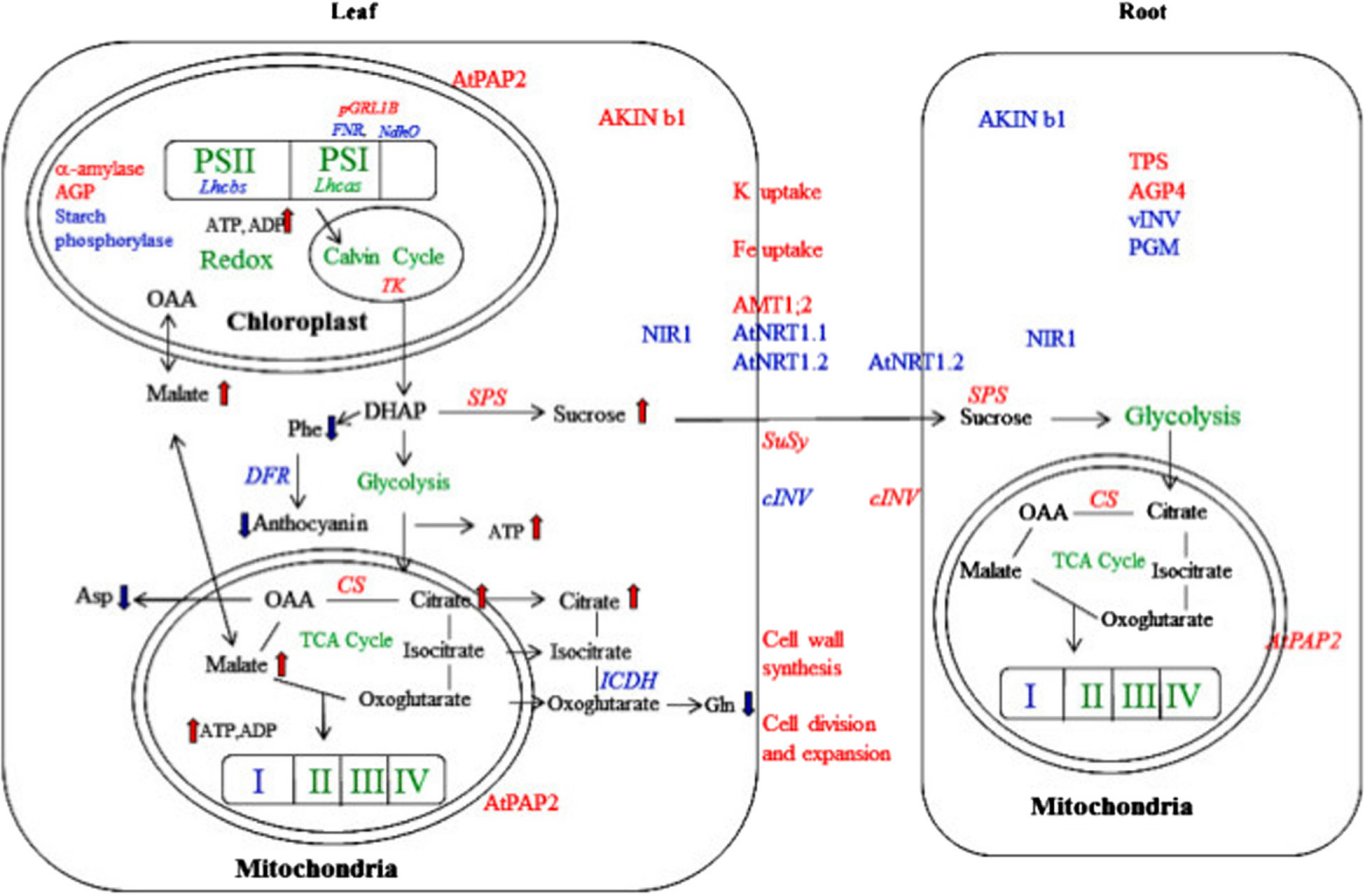
**Summary of the microarray data in leaves and roots.** Transcripts (italic) up-regulated and down-regulated in AtPAP2 OE plants are indicated in red and blue colors, respectively. Metabolites increased or decreased are indicated by red and blue arrows, respectively. Pathways in green means “not significantly changed”. I-IV, mitochondria complex I-IV; AKINb1, β1 subunit of SNRK; AGP, ADP-glucose pyrophoshorylase; cINV, cell wall invertase; CS, citrate synthase; DFR, dihydroflavonol 4-reductase; DHAP, dihydroxyacetone phosphate; ICDH, isocitrate dehydrogenase; OAA, oxaloacetate; PGM, phosphoglucomutase; PSI, photosystem I; PSII, photosystem II; SuSy, sucrose synthase; SPS, sucrose phosphate synthase; TK, transketolase; TPS, trehalose-6-phosphate synthase; vINV, vacuolar invertase.

The high ATP and sucrose contents of the OE lines must be generated by a higher output from the photosystems. Plant harvests light energy by PS I and PS II. In the leaves of both OE lines, while the mRNA transcripts of the genes of PS I and PS II core proteins and LHCI did not change significantly, many transcripts of the mobile LHCII components were altered (Additional file 8a). How could overexpression of AtPAP2 cause changes in the expressions of *Lhcb* genes? A possible explanation is that AtPAP2 overexpression triggers the regulation of redox-dependent retrograde signaling [31]. The expression of *Lhcb* genes are regulated by the redox state of the plastoquinone (PQ) pool [32]. PQ reduction suppresses *Lhcb* family gene expression to avoid absorption of excess light energy [31]. In addition, *Lhcb* genes could also be repressed by high sugar levels [33]. In addition to *Lhcb* genes, the upregulation of *PGRL1B*, a key component of the PGRL1-dependent CEF supercomplex [34] and the downregulation of *FNR2* were also significant, whether these changes can lead to a higher output of ATP from the photosystems would be an interesting subject for future studies (Figure 5). Photosynthesis also supplies reducing powers and many biological pathways are redox-regulated. The transcription levels of all thioredoxins were not significantly changed in both leaves and roots, and among the many Fd- and Trx-regulated enzymes in chloroplasts, only the transcriptions of *NiR* and *G6PD4* were specifically down- and up-regulated in both leaves and roots, respectively. This is reasonable because the activities of these proteins can be instantly regulated by the redox status (e.g. availability of light) instead of transcriptional regulation, which is more time-consuming. Our data indicates that the activities of NiR and G6PD4 are subject to both redox and transcriptional regulations.

Generally, if there is a higher output of ATP from chloroplasts, the demand of ATP production in mitochondria would be less. The increase in organic acids in leaves is consistent with the changes observed in the transcriptome in this study. The transcript of the cytosolic ICDH (AT1G65930) was strongly suppressed in the leaves of OE lines, which could account for the high citrate content in the OE lines [35]. Previous studies have shown that alterations of carboxylic acids can lead to alterations in photosynthesis and enhanced growth [36-38]. One mechanism shown to operate on altering organic acids is an effect on stomatal aperture, and increased growth by 25% [36]. Furthermore the role of citrate and malate in signaling changes in the transcriptome has been recently elucidated [39], showing interactions with hormone biosynthetic pathways such as gibberellin biosynthesis. Thus, overall the changes observed appear to mimic a reduction in carbon flow through the TCA cycle, which leads to an increase in sucrose and photosynthesis. Furthermore the changes due to increased levels of citrate, interact with hormone, ion (Fe^2+^ and Ca^2+^) and biotic defense pathways [39].

TCA metabolites are the substrates of various biomolecules. The OE lines contain a lower level of aspartate family amino acids (Asn, Asp, Lys, Met, Thr) than the WT, which could be caused by a higher capacity for malate production in the chloroplast. If excess malate is produced at the expense of OAA, the sole precursor of Asp family amino acids, and leads to a lower level of precursors of these amino acids. All of the above correlates with the results of metabolites analysis [6].

The fast-growing phenotypes of the AtPAP2 OE lines are dependent on the targeting of AtPAP2 to chloroplasts and mitochondria. While there are significant changes in energy harvesting and conversion processes related to chloroplasts’ functions (photosystem, starch and sucrose metabolism), the expression of most genes involved in catabolism, including glycolysis, TCA cycle and mitochondria respiratory chain, are unaltered in the OE lines. Similarly, the activities of many enzymes in these pathways are regulated by the energy status (e.g. ATP/ADP ratio, citrate, etc.) through allosteric regulation. Our data indicated that transcriptional regulation is not a major control mechanism of these pathways in the current study. Higher levels of energy production in chloroplasts would also alleviate the need for oxidative phosphorylation in mitochondria, which might cause the downregulation of the gene expression of Complex I components (Additional file 10). It would be interesting to measure the activity of Complex I in the OE lines. Reduced activity of Complex I may lower the rate of consumption of carbon, allocating more carbon to be used for anabolism and growth.

Leaves and roots are the source and sink of sucrose and energy and thus their carbon flows are different. Since the leaves of OE lines produced more sucrose, the supply of sucrose to roots would be increased. The changes in transcription profiles in the roots of OE lines thus likely reflect the impacts of higher sucrose supply. Cell wall invertase (*cwINV*) is a sucrose-cleavage enzyme and is responsible for hydrolyzing apoplastic sucrose. As shown in Figure 5, the transcription of a *cwINV* gene (AT3G13790) was downregulated in leaves but the transcription of another *cwINV* gene (AT3G13784, FC > 1.57 in OE21) was upregulated in roots. Furthermore, the transcriptions of two sucrose synthase *(SuSy)* genes were upregulated in leaves but not in roots. SuSy is a sucrose-cleavage enzyme which supplies hexose skeletons for cell wall synthesis. Their differential expression may affect the growth rates of leaves and roots. It is also true for the differential expression of certain genes of the starch synthesis pathway. Nonetheless, an *SPS* gene (*AtSPS2F*, AT5G11110) and a citrate synthase - like gene (AT2G11270) were upregulated in both leaves and roots, while a phosphoglucomutase (AT5G51820) was downregulated in both tissues.

Overall, the transcriptomic responses to AtPAP2 overexpression were consistent with the growth phenotypes and metabolite analysis [6]. A considerable amount of specific genes related to photosynthesis, sucrose metabolism, nitrogen metabolism and amino acid anabolism were significantly altered. A summary combining the transcriptome and metabolome for depicting the mechanisms responding to AtPAP2 overexpression is proposed (Figure 5). AtPAP2 overexpression may reprogram the photosystems and thus supply more ATP and carbon skeletons for sucrose and malate syntheses. The higher supply of malate causes the accumulation of organic acids, such as citrate and furmarate. The higher energy supply subsequently causes the alteration of many transcripts and metabolites.

## Conclusions

This study reported the global changes in transcriptome of source (leaves) and sink (roots) tissues when there are plenty supply of energy and sucrose. Overexpression of AtPAP2 enhances ATP production and sucrose synthesis in leaves, which provide more carbon skeleton for the roots. There are more than 30,000 genes in the genome of Arabidopsis and many gene families contain multiple members with highly homologous sequences or redundant functions. Our results reported the genes that are subject to transcriptional regulation when the energy status of the plant is elevated. Many scientists have attempted to enhance plant growth and yield by altering starch, sucrose, chloroplast or mitochondrial metabolism [40-43]. Other attempts included manipulation of transcription factors and hormones [44]. This study shows that alterations of other components feed into these pathways, and the identification of regulators or proteins that sense of mediate switches in metabolism offer an attractive avenue to increase biomass accumulation.

## Methods

### Plant materials and growth conditions

WT *Arabidopsis thaliana* ecotype Columbia (Col-0), an AtPAP2 T-DNA insertion mutant (Salk_013567) and two AtPAP2 OE lines (OE7 and OE21) were grown in soil under a 16-hr light (22°C)/8-hr dark (18°C) regime (long day, LD) at a light intensity of 120–150 μmol m^-2^ s^-1^[6].

### Determination of ATP and ADP by LC-MS/MS and bioluminescent assay

To extract ATP and ADP, 100 mg of leaves, freshly collected from 20-day-old plants at the middle of day, were ground in liquid nitrogen. 500 μL of 2.3% (w/v) trichloracetic acid was then added to the sample and the mixture was incubated on ice for 10 min. After centrifugation for 30 min at 16,000 g at 4°C, the supernatant (500 μL) was transferred to an ice-cold Eppendoff tube and the pH was adjusted to 7.0 by addition of 2.5 M K_2_CO_3_[8]. Measurments of ATP and ADP were carried out on a 3200 QTRAP LC-MS/MS System (AB Sciex, Foster City, USA) in negative mode [45]. To verify the LC-MS/MS results, ATP Bioluminescent Assay Kit (Sigma, FL-AA) was adopted [46]. The level of ATP was measured directly according to the kit’s protocol. To measure ADP, ADP was first converted into ATP by pyruvate kinase and ADP content = Total ATP after pyruvate kinase conversion – ATP before conversion. All data were analyzed by the statistical program SPSS Statistics 19.

### Microarray analysis

Leaves and roots were collected at the middle of the day and ground in liquid nitrogen. The roots were harvested from soil of 20-day-old Arabidopsis at the middle of day, and to avoid any interference caused by stresses or others, the harvest time did not exceed 1 hour. Total RNA extraction was performed using an RNeasy Mini Kit (Qiagen, USA) and quantified by the Bioanalyzer 2100 (Agilent Technologies, USA). First strand cDNA was synthesized using an oligo dT primer and 10 μg total RNA. NimbleGen Systems, Inc. (USA) performed the double stranded DNA synthesis and Cy 3 labeling from three biological replicates.

Normalized expression values were generated using a standard quantile normalization matrix [47] and the robust multichip average (RMA) algorithm [48], resulting in a final data set of 30361 probe identifiers in the leaf and 37118 in the root. The signal-to-noise-ratio for each spot was greater than 2.6. ArrayStar 3.0 (DNASTAR, USA) was used to draw heatmaps. The log-transformed data were subsequently analyzed for differential expression of genes between AtPAP2 OE lines and WT using the paired Student’s *t* test [49]. GO annotation was carried out with the GO terms of the TAIR database (http://www.Arabidopsis.org/tools/bulk/go/index.jsp) and the corresponding Arabidopsis gene locus identifiers were mapped to the Kyoto Encyclopedia of Genes and Genomes (KEGG) pathways (http://www.genome.jp/ kegg/) using the KegArray tool (Version 1.2.1). The percent (%) of significantly changed genes in each TAIR annotated category was calculated as follows: percent = the number of significantly changed genes divided by N × 100, where N represents the total number of genes annotated in each ontology. Identified genes were subsequently mapped to the MapMan databases (http://www.gabipd.org/projects/MapMan/). The microarray data in this work are deposited at GEO (http://www.ncbi.nlm.nih.gov/geo/) with the accession number: GSE40307.

### Quantitative real-time RT-PCR

Quantitative real-time RT-PCR analysis was carried out using cDNA samples transcribed from 20-day-old leaves of Arabidopsis. Primer3 Plus (http://www.bioinformatics.nl/cgi-bin/primer3plus/ primer3plus.cgi) was used to design the real-time RT-PCR primers. The PCR reactions were performed in a 20 μL volume containing a 2 × SYBR Green Master Mix (ABI systems), 50 ng cDNA, and 0.4 μM of forward and reverse primers in an ABI CFX96 thermocycler. The amplification parameters were 95°C for 1 min; followed by 40–50 cycles of 95°C, 15 s and 61°C, 30 s. β-actin was used as the internal control. For every transcript, each cDNA sample was analyzed in triplicate, and relative transcript abundance was calculated by normalizing to the maximum level. The comparative Ct method was used to calculate the relative gene expression levels across the samples. The relative expression level of each gene in one sample (ΔCt) was calculated as follows: Ct target gene – Ct beta-actin. The relative expression of each gene in two different samples (ΔΔCt) was calculated as follows: ΔCt (sample 1) –ΔCt (sample 2). The primers used are shown in Additional file 13.

### Sucrose treatment and anthocyanin measurement

Sucrose gradients from 0% to 15% (w/v) were employed to test the post-germination growth of Arabidopsis seedlings. Five–day-old seedlings grown on normal MS medium (2%, w/v, sucrose) were transferred to MS medium with different concentration of sucrose for 3 days. For mannitol and sorbitol (8% and 9%; w/v) treatments, these sugars were added to the MS medium with 1% (w/v) sucrose. Anthocyanin content of seedlings was determined spectroscopically as described [50]. RT-PCR analysis was carried out according to Teng [51].

## Competing interests

The authors declare that they have no competing interests.

## Authors’ contributions

FS carried out experimental design, sample collection, microarray data integration, anthocyanin measurement and drafted the manuscript, CL participated in the microarray data analysis and performed the ATP measurement experiments, JW, JY and PZ participated in the microarray data analysis and provided helpful suggestions, and BL was responsible for the overall concept, experimental design, data analysis, and revising manuscript. All authors read and approved the manuscript.

## Supplementary Material

Additional file 1 Leaf microarray data. Click here for file

Additional file 2 Root microarray data. Click here for file

Additional file 3 **MapMan diagram of genes involved in sucrose synthesis and photosynthesis.** Gene transcription significantly up- and downregulated (1.5-fold change and P < 0.05) in the leaves (A, B) and roots (C, D) of OE lines are indicated in red and blue, respectively. Scale bars display log2 fold changes. Click here for file

Additional file 4 MapMan diagram of genes involved in regulation in Leaves (A) and Roots (B). Click here for file

Additional file 5 MapMan diagram of genes associated with mitochondrial electron transport in Leaves (A) and Roots (B). Click here for file

Additional file 6 MapMan diagram of genes of transcription factors in Leaves (A) and Roots (B). Click here for file

Additional file 7 MapMan diagram of of genes associated with biotic stresses in Leaves (A) and Roots (B). Click here for file

Additional file 8 Functional categories (Leaf). Click here for file

Additional file 9 Functional categories (Root). Click here for file

Additional file 10 Respiratory chain in mitochondria. Click here for file

Additional file 11 Transcription factors. Click here for file

Additional file 12 Nucleus-encoded chloroplastic and mitochondrial proteins in leaves and roots. Click here for file

Additional file 13 RT-PCR Primers. Click here for file

Additional file 14 **Validation of leaf microarray data by real-time RT-PCR.** Columns in white and black indicate microarray and real-time RT-PCR data, respectively. Click here for file

Additional file 15 **Induction of anthocyanin by sucrose.** Five-day-old seedlings were transferred to MS medium containing different concentrations of sucrose for another 3 days. Click here for file

Additional file 16 **Anthocyanin levels in WT, AtPAP2 T-DNA and AtPAP2 OE lines after sucrose (a) and osmotic sugar (b) treatment.** Five-day-old seedlings were transferred to sucrose gradient MS medium for additional 3 days. Anthocyanin level was measured. Click here for file

Additional file 17 **RT-PCR analysis of genes after sucrose treatment.** Five-day-old seedlings were transferred to MS medium (0%, 2.5%, 6% sucrose, w/v) for 3 days before RT-PCR analysis. Elongation factor (*EF*) was taken as a control. Click here for file

## References

[B1] Schenk G Guddat LW Ge Y Carrington LE Hume DA Hamilton S de Jersey J Identification of mammalian-like purple acid phosphatases in a wide range of plants Gene 2000 250 1–2 117 125 10854785 10.1016/s0378-1119(00)00186-4

[B2] Lung SC Leung A Kuang R Wang Y Leung P Lim BL Phytase activity in tobacco (*Nicotiana tabacum*) root exudates is exhibited by a purple acid phosphatase Phytochemistry 2008 69 2 365 373 10.1016/j.phytochem.2007.06.036 17897689

[B3] Kuang RB Chan KH Yeung E Lim BL Molecular and biochemical characterization of AtPAP15, a purple acid phosphatase with phytase activity, in Arabidopsis Plant Physiol 2009 151 1 199 209 10.1104/pp.109.143180 19633233 PMC2735976

[B4] Sun F Carrie C Law S Murcha MW Zhang R Law YS Suen PK Whelan J Lim BL AtPAP2 is a tail-anchored protein in the outer membrane of chloroplasts and mitochondria Plant Signal Behav 2012 7 8 927 932 10.4161/psb.20769 22751362 PMC3474687

[B5] Derelle E Ferraz C Rombauts S Rouze P Worden AZ Robbens S Partensky F Degroeve S Echeynie S Cooke R Genome analysis of the smallest free-living eukaryote Ostreococcus tauri unveils many unique features Proc Natl Acad Sci 2006 103 31 11647 11652 10.1073/pnas.0604795103 16868079 PMC1544224

[B6] Sun F Suen PK Zhang Y Liang C Carrie C Whelan J Ward JL Hawkins ND Jiang L Lim BL A dual-targeted purple acid phosphatase in *Arabidopsis thaliana* moderates carbon metabolism and its overexpression leads to faster plant growth and higher seed yield New Phytol 2012 194 1 206 219 10.1111/j.1469-8137.2011.04026.x 22269069

[B7] Zhang YJ Yu L Yung KF Leung DYC Sun F Lim BL Over-expression of AtPAP2 in *Camelina sativa* leads to faster plant growth and higher seed yield Biotechnol Biofuels 2012 5 19 10.1186/1754-6834-5-19 22472516 PMC3361479

[B8] Meyer EH Tomaz T Carroll AJ Estavillo G Delannoy E Tanz SK Small ID Pogson BJ Millar AH Remodeled respiration in *ndufs4* with low phosphorylation efficiency suppresses Arabidopsis germination and growth and alters control of metabolism at night Plant Physiol 2009 151 2 603 619 10.1104/pp.109.141770 19675153 PMC2754622

[B9] Thimm O Blasing O Gibon Y Nagel A Meyer S Kruger P Selbig J Muller LA Rhee SY Stitt M MAPMAN: a user-driven tool to display genomics data sets onto diagrams of metabolic pathways and other biological processes Plant J 2004 37 6 914 939 10.1111/j.1365-313X.2004.02016.x 14996223

[B10] Voss I Goss T Murozuka E Altmann B McLean KJ Rigby SEJ Munro AW Scheibe R Hase T Hanke GT FdC1, a novel ferredoxin protein capable of alternative electron partitioning, increases in conditions of acceptor limitation at Photosystem I J Biol Chem 2011 286 1 50 59 10.1074/jbc.M110.161562 20966083 PMC3013009

[B11] Peng LW Shikanai T Supercomplex formation with photosystem I is required for the stabilization of the chloroplast NADH dehydrogenase-like complex in Arabidopsis Plant Physiol 2011 155 4 1629 1639 10.1104/pp.110.171264 21278308 PMC3091109

[B12] Howe CJ Schlarb-Ridley BG Wastl J Purton S Bendall DS The novel cytochrome c6 of chloroplasts: a case of evolutionary bricolage? J Exp Bot 2006 57 1 13 22 16317035 10.1093/jxb/erj023

[B13] Kherraz K Kameli A Homology modeling of ferredoxin-nitrite reductase from *Arabidopsis thaliana* Bioinformation 2011 6 3 115 119 10.6026/97320630006115 21584187 PMC3089885

[B14] Schurmann P Buchanan BB The ferredoxin/thioredoxin system of oxygenic photosynthesis Antioxid Redox Signal 2008 10 7 1235 1273 10.1089/ars.2007.1931 18377232

[B15] Lou Y Gou JY Xue HW PIP5K9, an Arabidopsis phosphatidylinositol monophosphate kinase, interacts with a cytosolic invertase to negatively regulate sugar-mediated root growth Plant Cell 2007 19 1 163 181 10.1105/tpc.106.045658 17220200 PMC1820962

[B16] Liu KH Tsay YF Switching between the two action modes of the dual-affinity nitrate transporter CHL1 by phosphorylation EMBO J 2003 22 5 1005 1013 10.1093/emboj/cdg118 12606566 PMC150351

[B17] Huang NC Liu KH Lo HJ Tsay YF Cloning and functional characterization of an Arabidopsis nitrate transporter gene that encodes a constitutive component of low-affinity uptake Plant Cell 1999 11 8 1381 1392 10449574 10.1105/tpc.11.8.1381 PMC144300

[B18] Yoshiba Y Kiyosue T Katagiri T Ueda H Mizoguchi T Yamaguchishinozaki K Wada K Harada Y Shinozaki K Correlation between the induction of a gene for delta(1)-pyrroline-5-carboxylate synthetase and the accumulation of proline in *Arabidopsis thaliana* under osmotic stress Plant J 1995 7 5 751 760 10.1046/j.1365-313X.1995.07050751.x 7773306

[B19] Tzin V Galili G The biosynthetic pathways for shikimate and aromatic amino acids in *Arabidopsis thaliana* Arabidopsis Book 2010 8 e0132 22303258 10.1199/tab.0132 PMC3244902

[B20] Hodges M Flesch V Galvez S Bismuth E Higher plant NADP(+)-dependent isocitrate dehydrogenases, ammonium assimilation and NADPH production Plant Physiol Biochem 2003 41 6–7 577 585

[B21] Lee SC Lan WZ Kim BG Li LG Cheong YH Pandey GK Lu GH Buchanan BB Luan S A protein phosphorylation/dephosphorylation network regulates a plant potassium channel Proc Natl Acad Sci 2007 104 40 15959 15964 10.1073/pnas.0707912104 17898163 PMC2000415

[B22] Jiao YL Yang HJ Ma LG Sun N Yu HY Liu T Gao Y Gu HY Chen ZL Wada M A genome-wide analysis of blue-light regulation of Arabidopsis transcription factor gene expression during seedling development Plant Physiol 2003 133 4 1480 1493 10.1104/pp.103.029439 14605227 PMC300705

[B23] Riano-Pachon DM Ruzicic S Dreyer I Mueller-Roeber B PlnTFDB: an integrative plant transcription factor database BMC Bioinforma 2007 8 42 10.1186/1471-2105-8-42 PMC1802092 17286856

[B24] Li WX Oono Y Zhu JH He XJ Wu JM Iida K Lu XY Cui XP Jin HL Zhu JK The Arabidopsis NFYA5 transcription factor is regulated transcriptionally and posttranscriptionally to promote drought resistance Plant Cell 2008 20 8 2238 2251 10.1105/tpc.108.059444 18682547 PMC2553615

[B25] Zhou JL Lee CH Zhong RQ Ye ZH MYB58 and MYB63 are transcriptional activators of the lignin biosynthetic pathway during secondary cell wall formation in Arabidopsis Plant Cell 2009 21 1 248 266 10.1105/tpc.108.063321 19122102 PMC2648072

[B26] Ito S Song YH Josephson-Day AR Miller RJ Breton G Olmstead RG Imaizumi T FLOWERING BHLH transcriptional activators control expression of the photoperiodic flowering regulator CONSTANS in Arabidopsis Proc Natl Acad Sci 2012 109 9 3582 3587 10.1073/pnas.1118876109 22334645 PMC3295255

[B27] Kleffmann T Russenberger D von Zychlinski A Christopher W Sjolander K Gruissem W Baginsky S The *Arabidopsis thaliana* chloroplast proteome reveals pathway abundance and novel protein functions Curr Biol 2004 14 5 354 362 10.1016/j.cub.2004.02.039 15028209

[B28] Duncan O Taylor NL Carrie C Eubel H Kubiszewski-Jakubiak S Zhang BT Narsai R Millar AH Whelan J Multiple lines of evidence localize signaling, morphology, and lipid biosynthesis machinery to the mitochondrial outer membrane of Arabidopsis Plant Physiol 2011 157 3 1093 1113 10.1104/pp.111.183160 21896887 PMC3252152

[B29] Heazlewood JL Tonti-Filippini JS Gout AM Day DA Whelan J Millar AH Experimental analysis of the Arabidopsis mitochondrial proteome highlights signaling and regulatory components, provides assessment of targeting prediction programs, and indicates plant-specific mitochondrial proteins Plant Cell 2004 16 1 241 256 10.1105/tpc.016055 14671022 PMC301408

[B30] Solfanelli C Poggi A Loreti E Alpi A Perata P Sucrose-specific induction of the anthocyanin biosynthetic pathway in Arabidopsis Plant Physiol 2006 140 2 637 646 10.1104/pp.105.072579 16384906 PMC1361330

[B31] Fey V Wagner R Brautigam K Wirtz M Hell R Dietzmann A Leister D Oelmuller R Pfannschmidt T Retrograde plastid redox signals in the expression of nuclear genes for chloroplast proteins of *Arabidopsis thaliana* J Biol Chem 2005 280 17 17572 10.1074/jbc.M406358200 15561727

[B32] Nott A Jung HS Koussevitzky S Chory J Plastid-to-nucleus retrograde signaling Annu Rev Plant Biol 2006 57 739 759 10.1146/annurev.arplant.57.032905.105310 16669780

[B33] Jang JC Sheen J Sugar sensing in higher plants Trends Plant Sci 1997 2 6 208 214 10.1016/S1360-1385(97)89545-3 PMC160552 7827498

[B34] DalCorso G Pesaresi P Masiero S Aseeva E Schunemann D Finazzi G Joliot P Barbato R Leister D A complex containing PGRL1 and PGR5 is involved in the switch between linear and cyclic electron flow in Arabidopsis Cell 2008 132 2 273 285 10.1016/j.cell.2007.12.028 18243102

[B35] Sulpice R Sienkiewicz-Porzucek A Osorio S Krahnert I Stitt M Fernie AR Nunes-Nesi A Mild reductions in cytosolic NADP-dependent isocitrate dehydrogenase activity result in lower amino acid contents and pigmentation without impacting growth Amino Acids 2010 39 4 1055 1066 10.1007/s00726-010-0617-0 20473773 PMC2945463

[B36] Araujo WL Nunes-Nesi A Nikoloski Z Sweetlove LJ Fernie AR Metabolic control and regulation of the tricarboxylic acid cycle in photosynthetic and heterotrophic plant tissues Plant Cell and Environ 2012 35 1 21 10.1111/j.1365-3040.2011.02332.x 21477125

[B37] Carrari F Nunes-Nesi A Gibon Y Lytovchenko A Loureiro ME Fernie AR Reduced expression of aconitase results in an enhanced rate of photosynthesis and marked shifts in carbon partitioning in illuminated leaves of wild species tomato Plant Physiol 2003 133 1322 1335 10.1104/pp.103.026716 14551334 PMC281627

[B38] Morgan MJ Osorio S Gehl B Baxter CJ Kruger NJ Ratcliffe RG Fernie AR Sweetlove LJ Metabolic engineering of tomato fruit organic acid content guided by biochemical analysis of an introgression line Plant Physiol 2013 161 397 407 10.1104/pp.112.209619 23166354 PMC3532270

[B39] Finkemeier I Konig AC Heard W Nunes-Nesi A Pham PA Leister D Fernie AR Sweetlove LJ Transcriptomic analysis of the role of carboxylic acids in metabolite signaling in Arabidopsis leaves Plant Physiol 2013 162 239 253 10.1104/pp.113.214114 23487434 PMC3641205

[B40] Nunes-Nesi A Carrari F Lytovchenko A Smith AMO Loureiro ME Ratcliffe RG Sweetlove LJ Fernie AR Enhanced photosynthetic performance and growth as a consequence of decreasing mitochondrial malate dehydrogenase activity in transgenic tomato plants Plant Physiol 2005 137 2 611 622 10.1104/pp.104.055566 15665243 PMC1065362

[B41] Araujo WL Nunes-Nesi A Osorio S Usadel B Fuentes D Nagy R Balbo I Lehmann M Studart-Witkowski C Tohge T Antisense inhibition of the iron-sulphur subunit of succinate dehydrogenase enhances photosynthesis and growth in tomato via an organic acid-mediated effect on stomatal aperture Plant Cell 2011 23 2 600 627 10.1105/tpc.110.081224 21307286 PMC3077794

[B42] Baxter CJ Foyer CH Turner J Rolfe SA Quick WP Elevated sucrose-phosphate synthase activity in transgenic tobacco sustains photosynthesis in older leaves and alters development J Exp Bot 2003 54 389 1813 1820 10.1093/jxb/erg196 12815030

[B43] Sonnewald U Hajirezaei MR Kossmann J Heyer A Trethewey RN Willmitzer L Increased potato tuber size resulting from apoplastic expression of a yeast invertase Nat Biotechnol 1997 15 8 794 797 10.1038/nbt0897-794 9255797

[B44] Gonzalez N Beemster GTS Inze D David and Goliath: what can the tiny weed Arabidopsis teach us to improve biomass production in crops? Curr Opin Plant Biol 2009 12 2 157 164 10.1016/j.pbi.2008.11.003 19119056

[B45] Luo B Groenke K Takors R Wandrey C Oldiges M Simultaneous determination of multiple intracellular metabolites in glycolysis, pentose phosphate pathway and tricarboxylic acid cycle by liquid chromatography-mass spectrometry J Chromatogr A 2007 1147 2 153 164 10.1016/j.chroma.2007.02.034 17376459

[B46] Ford SR Leach FR Bioluminescent assay of the adenylate energy charge Methods Mol Biol 1998 102 69 81 9680610 10.1385/0-89603-520-4:69

[B47] Bolstad BM Irizarry RA Astrand M Speed TP A comparison of normalization methods for high density oligonucleotide array data based on variance and bias Bioinformatics 2003 19 2 185 193 10.1093/bioinformatics/19.2.185 12538238

[B48] Irizarry RA Bolstad BM Collin F Cope LM Hobbs B Speed TP Summaries of affymetrix GeneChip probe level data Nucleic Acids Res 2003 31 4 e15 10.1093/nar/gng015 12582260 PMC150247

[B49] Benjamini Y Hochberg Y Controlling the false discovery rate - a practical and powerful approach to multiple testing J R Stat Soc Series B 1995 57 1 289 300

[B50] Mita S Murano N Akaike M Nakamura K Mutants of *Arabidopsis thaliana* with pleiotropic effects on the expression of the gene for beta-amylase and on the accumulation of anthocyanin that are inducible by sugars Plant J 1997 11 4 841 851 10.1046/j.1365-313X.1997.11040841.x 9161039

[B51] Teng S Keurentjes J Bentsink L Koornneef M Smeekens S Sucrose-specific induction of anthocyanin biosynthesis in Arabidopsis requires the MYB75/PAP1 gene Plant Physiol 2005 139 4 1840 1852 10.1104/pp.105.066688 16299184 PMC1310563

