# Supplementary material for: Global transcriptome analysis of AtPAP2 - overexpressing Arabidopsisthaliana with elevated ATP
Source: BMC Genomics. 2013 Nov 1;14:752. doi: 10.1186/1471-2164-14-752 (PMC3829102; doi:10.1186/1471-2164-14-752)
Supplement: Additional file 3 — MapMan diagram of genes involved in sucrose synthesis and photosynthesis. Gene transcription significantly up- and downregulated (1.5-fold change and P < 0.05) in the leaves (A, B) and roots (C, D) of OE lines are indicated in red and blue, respectively. Scale bars display log2 fold changes. [file 1471-2164-14-752-S3.pdf]

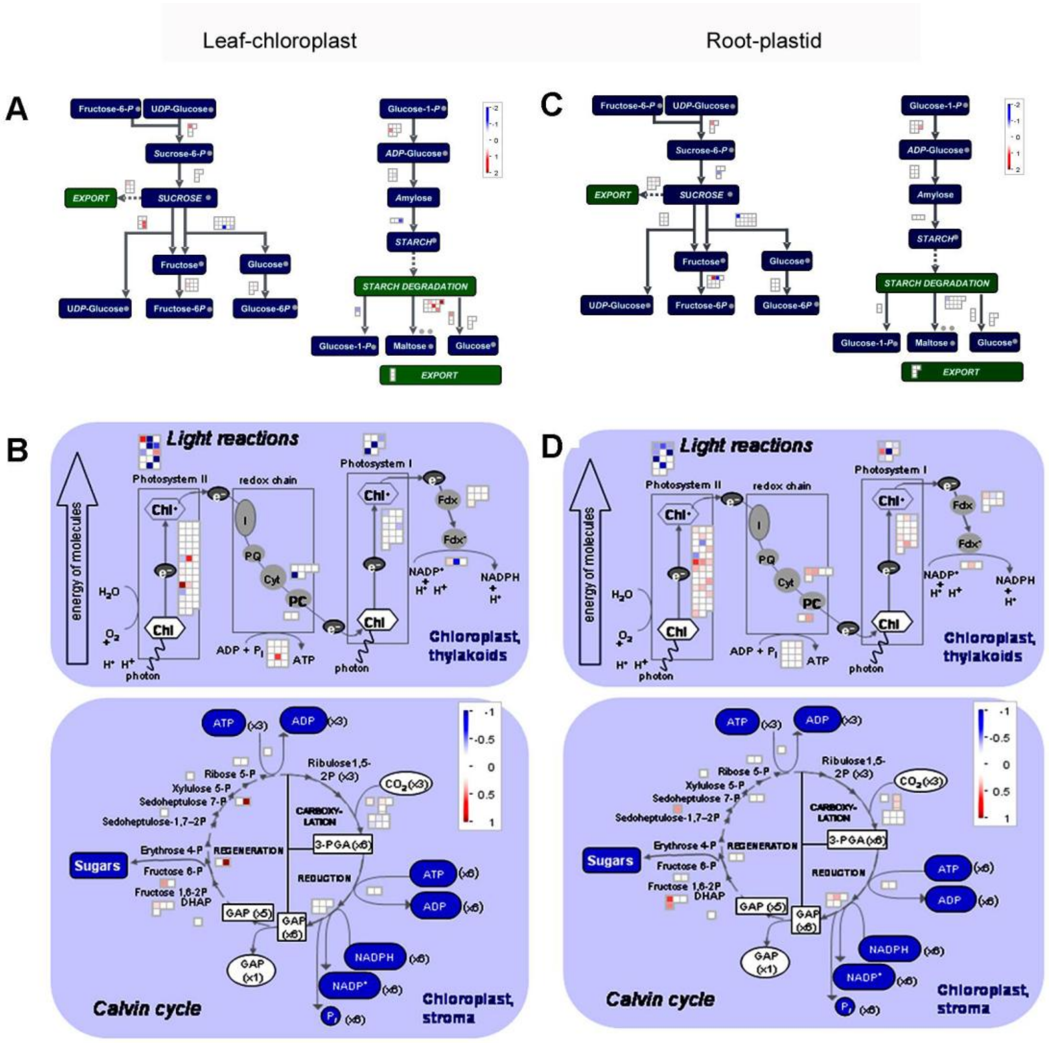

Additional file 3: MapMan diagram of genes involved in sucrose synthesis and photosynthesis. Gene transcription significantly up- and downregulated (1.5 fold change and P<0.05) in the leaves (A, B) and roots (C, D) of OE lines are indicated in red and blue, respectively. Scale bars display log2 fold changes.
