# Supplementary material for: Global transcriptome analysis of AtPAP2 - overexpressing Arabidopsisthaliana with elevated ATP
Source: BMC Genomics. 2013 Nov 1;14:752. doi: 10.1186/1471-2164-14-752 (PMC3829102; doi:10.1186/1471-2164-14-752)
Supplement: Additional file 4 — MapMan diagram of genes involved in regulation in Leaves (A) and Roots (B). [file 1471-2164-14-752-S4.pdf]

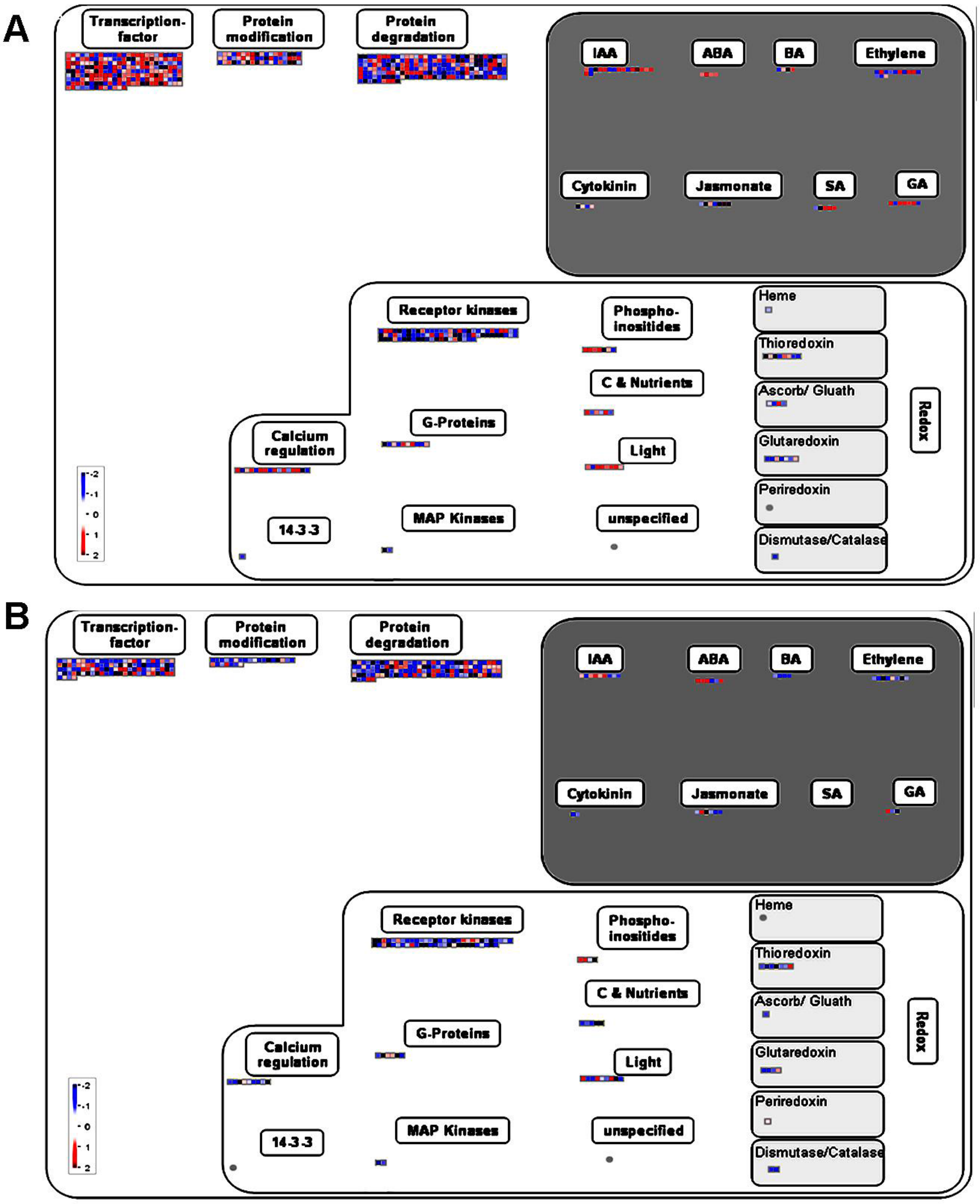

Additional file 4: MapMan diagram of genes involved in regulation in leaves (A) and roots (B). Genes significantly up-and downregulated (1.5 fold change and P<0.05) in OE lines are indicated in red and blue, respectively. Scale bars display log2 fold changes.
