# Supplementary figures and images for: Global transcriptome analysis of AtPAP2 - overexpressing Arabidopsisthaliana with elevated ATP
Source: BMC Genomics. 2013 Nov 1;14:752. doi: 10.1186/1471-2164-14-752 (PMC3829102; doi:10.1186/1471-2164-14-752)

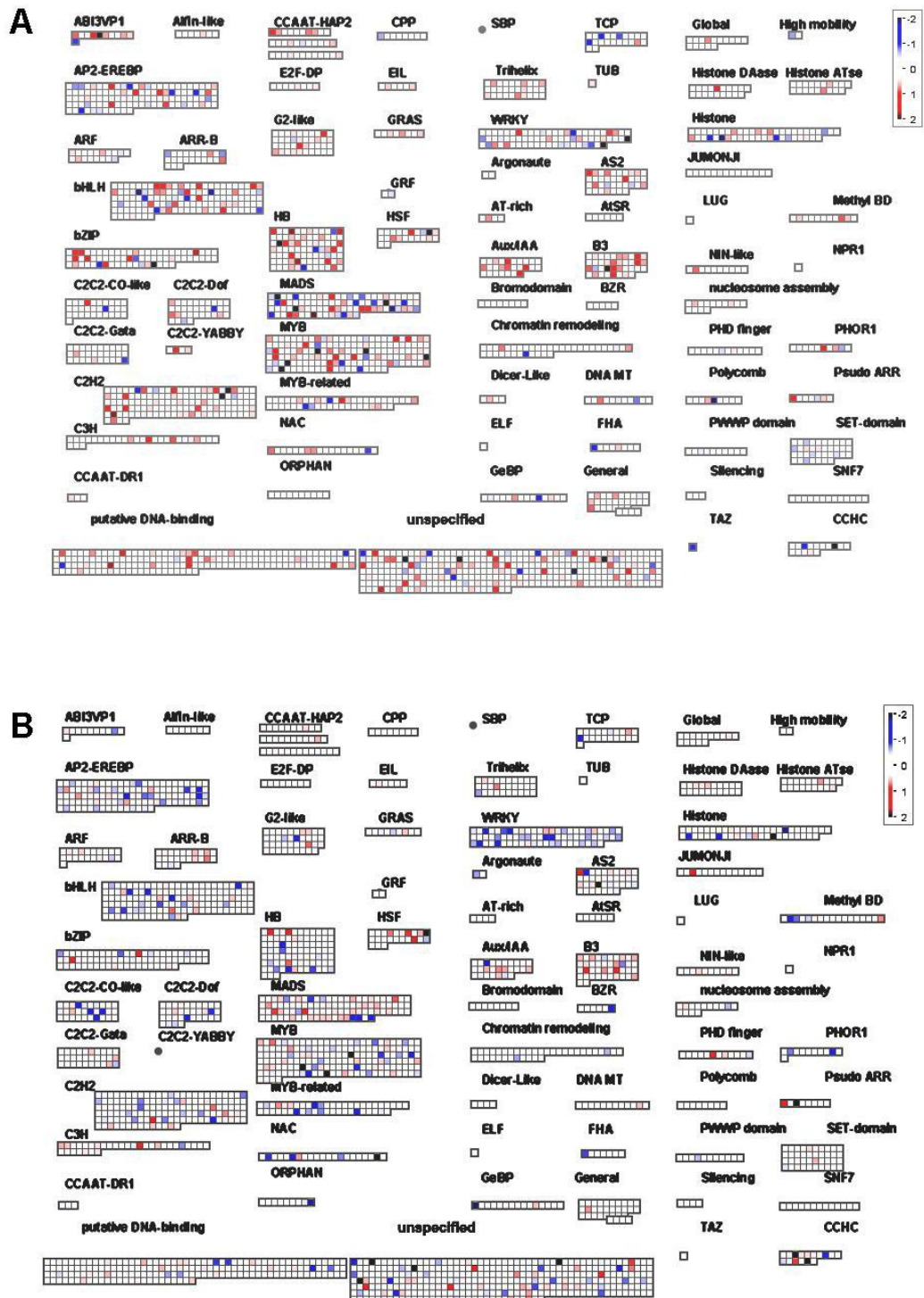

Supplement: Additional file 6 — MapMan diagram of genes of transcription factors in Leaves (A) and Roots (B). [file 1471-2164-14-752-S6.pdf]
