# Supplementary material for: Global transcriptome analysis of AtPAP2 - overexpressing Arabidopsisthaliana with elevated ATP
Source: BMC Genomics. 2013 Nov 1;14:752. doi: 10.1186/1471-2164-14-752 (PMC3829102; doi:10.1186/1471-2164-14-752)
Supplement: Additional file 13 — RT-PCR Primers. [file 1471-2164-14-752-S13.pdf]

Additional file 13. Lists of primers used for real-time RT-PCR for validating microarray data.

At3g60950	
Forward	ACAACAGCAAGGGAAAGGAACT
Reverse	AAGCAGCAAGAGAAACGAAAAG
At1g05000	
Forward	TGTGCCCGGAGCCCTACC
Reverse	CTTTCAGTGCCATGCGGATTTT
At5g24240	
Forward	AAGCCCATTGACGAGGAACC
Reverse	GGCGGAACACCAGCAAAAC
At1g28390	
Forward	GCCGCCGTCAAGAGAACAAC
Reverse	CTCCGGTGGTCAACGCAGTAA
At3g24660	
Forward	GTTGTTGTGGCCTCGTTGTTA
Reverse	CTTTCCTTCACCGCCTTCTTTC
Actin	
Forward	CCCGCTATGTATGTCGC
Reverse	AAGGTCAAGACGGAGGAT
