# Supplementary material for: Global transcriptome analysis of AtPAP2 - overexpressing Arabidopsisthaliana with elevated ATP
Source: BMC Genomics. 2013 Nov 1;14:752. doi: 10.1186/1471-2164-14-752 (PMC3829102; doi:10.1186/1471-2164-14-752)
Supplement: Additional file 14 — Validation of leaf microarray data by real-time RT-PCR. Columns in white and black indicate microarray and real-time RT-PCR data, respectively. [file 1471-2164-14-752-S14.pdf]

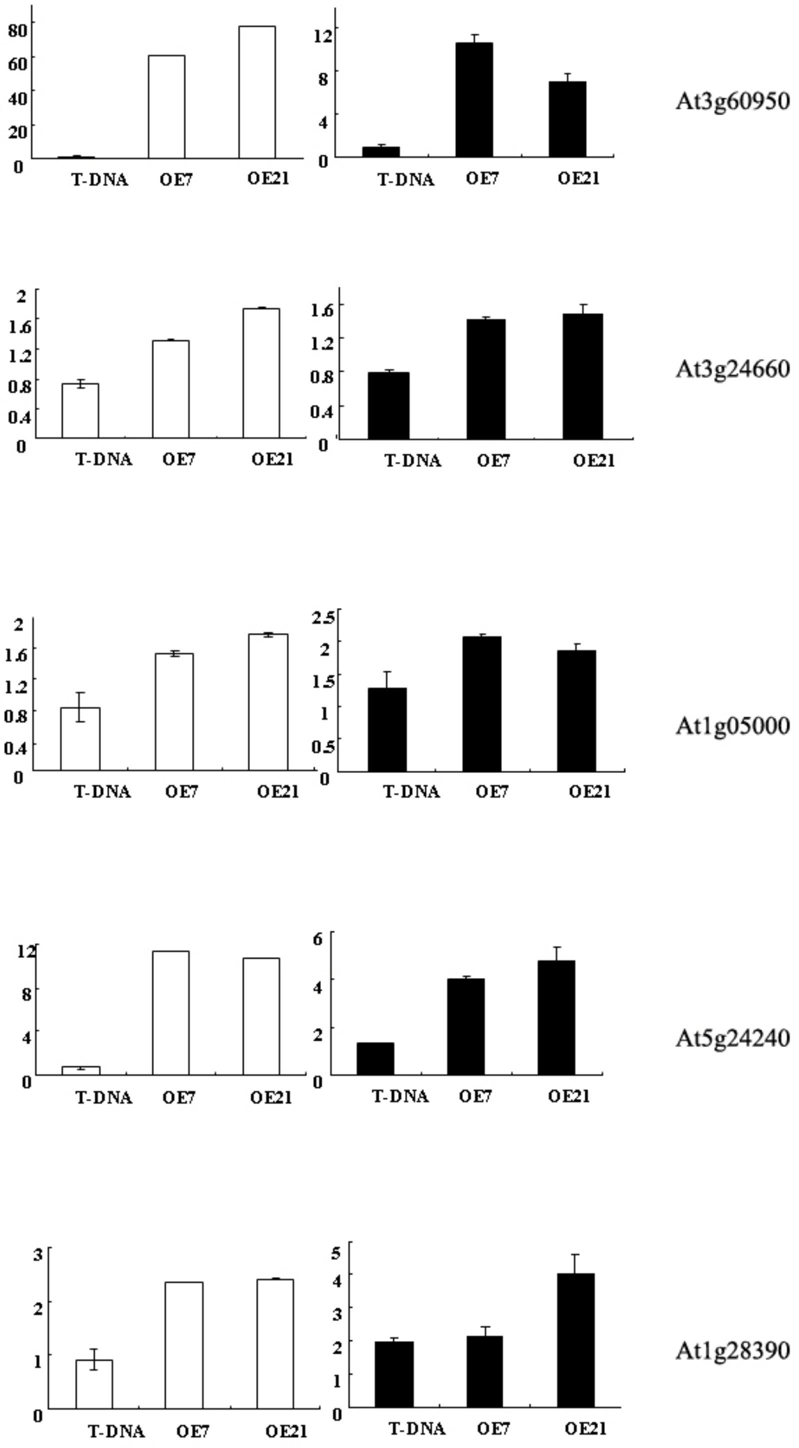

Additional file 14: Validation of leaf microarray data by real-time RT-PCR. Columns in white and black indicate microarray and real-time RT-PCR data, respectively.
