# Supplementary material for: Global transcriptome analysis of AtPAP2 - overexpressing Arabidopsisthaliana with elevated ATP
Source: BMC Genomics. 2013 Nov 1;14:752. doi: 10.1186/1471-2164-14-752 (PMC3829102; doi:10.1186/1471-2164-14-752)
Supplement: Additional file 15 — Induction of anthocyanin by sucrose. Five-day-old seedlings were transferred to MS medium containing different concentrations of sucrose for another 3 days. [file 1471-2164-14-752-S15.pdf]

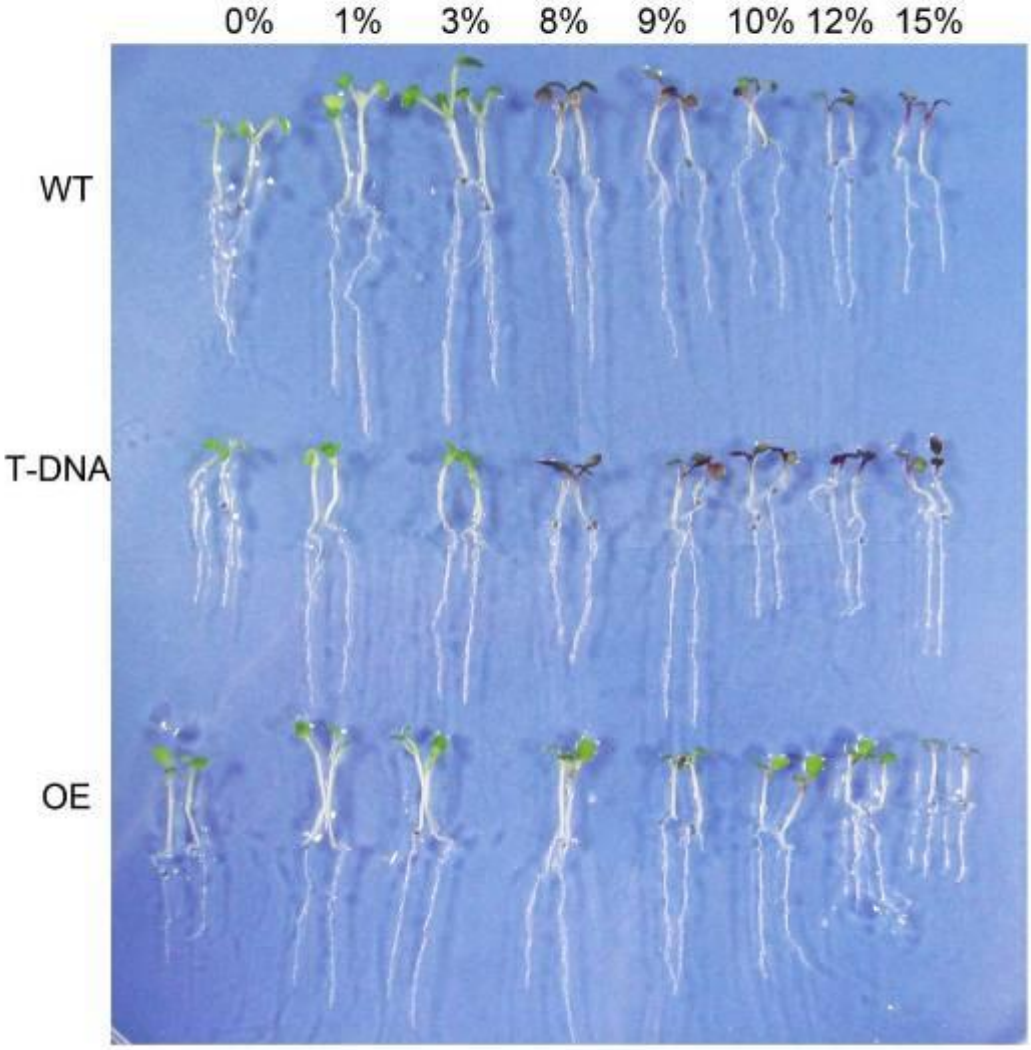


**Additional file 15: Induction of anthocyanin by sucrose.** Five-day-old seedlings were transferred to MS medium containing different concentrations of sucrose for another 3 days.
