# Supplementary material for: Global transcriptome analysis of AtPAP2 - overexpressing Arabidopsisthaliana with elevated ATP
Source: BMC Genomics. 2013 Nov 1;14:752. doi: 10.1186/1471-2164-14-752 (PMC3829102; doi:10.1186/1471-2164-14-752)
Supplement: Additional file 16 — Anthocyanin levels in WT, AtPAP2 T-DNA and AtPAP2 OE lines after sucrose (a) and osmotic sugar (b) treatment. Five-day-old seedlings were transferred to sucrose gradient MS medium for additional 3 days. Anthocyanin level was measured. [file 1471-2164-14-752-S16.pdf]

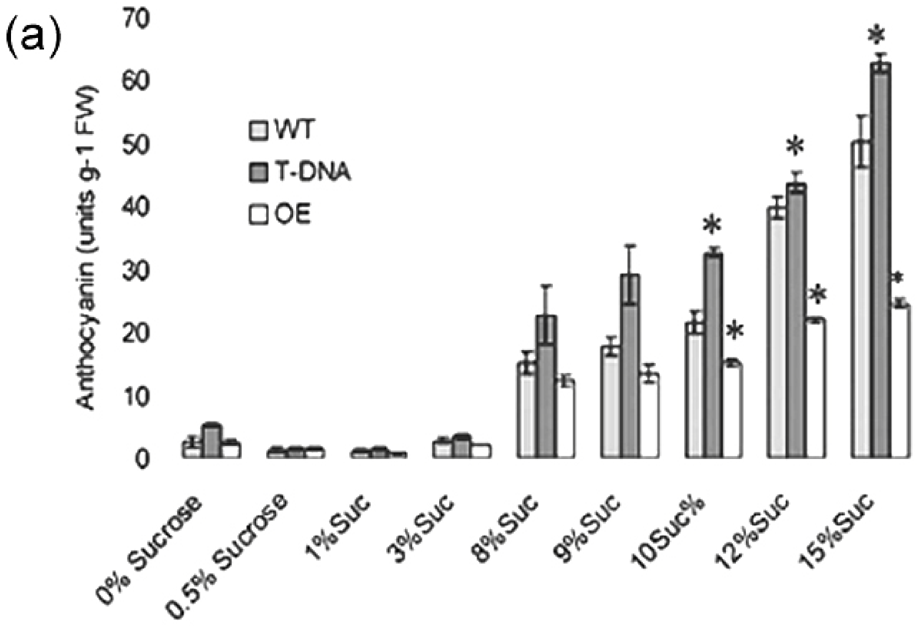

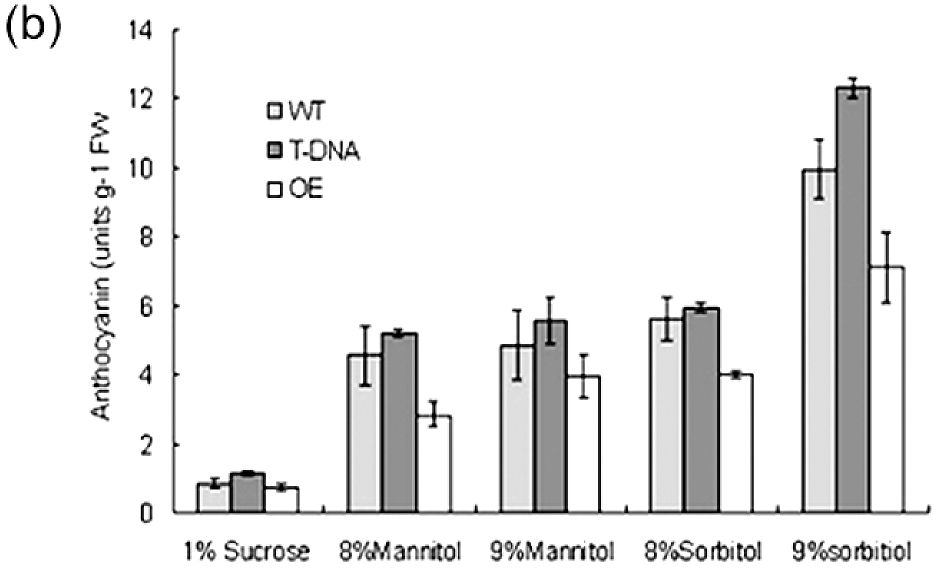

Additional file 16: Anthocyanin levels in WT, AtPAP2 T-DNA and AtPAP2 OE lines after sucrose treatment (a) and osmotic sugar treatment (b). Five-day-old seedlings were transferred to sucrose gradient MS medium for additional 3 days. Anthocyanin level was measured.
