# Supplementary material for: Global transcriptome analysis of AtPAP2 - overexpressing Arabidopsisthaliana with elevated ATP
Source: BMC Genomics. 2013 Nov 1;14:752. doi: 10.1186/1471-2164-14-752 (PMC3829102; doi:10.1186/1471-2164-14-752)
Supplement: Additional file 17 — RT-PCR analysis of genes after sucrose treatment. Five-day-old seedlings were transferred to MS medium (0%, 2.5%, 6% sucrose, w/v) for 3 days before RT-PCR analysis. Elongation factor (EF) was taken as a control. [file 1471-2164-14-752-S17.pdf]

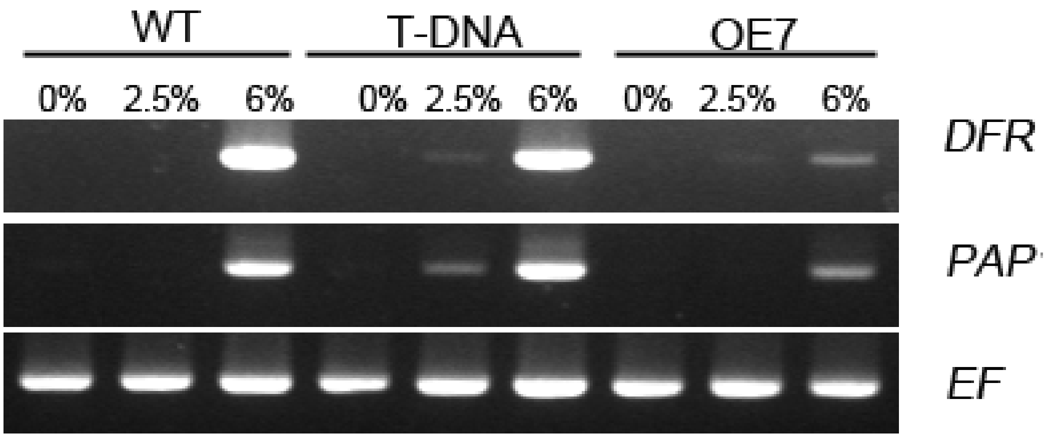

Additional file 17: RT-PCR analysis of DFR and PAP genes after sucrose treatment. Five-day-old seedlings were transferred to MS medium (0%, 2.5%, 6% sucrose, w/v) for 3 days before RT-PCR analysis. Elongation factor (*EF*) was taken as a control. PCR conditions are according to Teng [50].
